# Multi‐Organ Inflammatory and Endocrine Crosstalk Shapes the Early Brain Response to Combined Burn and Blast Injury

**DOI:** 10.1096/fj.202504681R

**Published:** 2026-10-29

**Authors:** Hana C. Schwierling, Satarupa Sengupta, John G. Noel, Christopher J. O'Connell, Andrew S. Potter, Mike Adam, Matthew J. Robson, Jason C. Gardner

**Affiliations:** ^1^ Division of Pharmaceutical Sciences, James L. Winkle College of Pharmacy University of Cincinnati Cincinnati Ohio USA; ^2^ Department of Internal Medicine University of Cincinnati College of Medicine Cincinnati Ohio USA; ^3^ Department of Genetics Harvard University School of Medicine Cambridge Massachusetts USA; ^4^ Cincinnati Children's Hospital Medical Center Cincinnati Ohio USA

**Keywords:** anemia, blast‐induced brain injury, burn injury, hypothalamic–pituitary–adrenal axis, interleukin‐1, neuroinflammation

## Abstract

The pathology of concomitant burn and traumatic brain injury remains poorly defined, despite their frequent co‐occurrence in military settings and the potential for deleterious synergy. To address this gap, we combined established models of blast‐induced mild traumatic brain injury and 15% total body surface area flame burn to approximate a common combat‐related trauma and examined how the injuries interact to shape systemic inflammation and brain transcriptional programs. Burn injury induced systemic G‐CSF and IL‐6 secretion and hematopoietic reprioritization, whereas overlapping brain injury did not substantially alter these systemic effects but magnified brain transcriptional responses relative to single injuries. Bulk RNA‐seq profiling at 6 h post‐injury revealed enriched gene sets associated with inflammation and endocrine stress in the combined injury group, alongside elevated systemic cytokines and corticosterone; thereby suggesting concurrent immune‐endocrine activation. Since our prior work demonstrated burn wound‐associated IL‐1 signaling induces G‐CSF and IL‐6 secretion, we tested IL‐1 neutralizing antibodies and anakinra as therapies. Treatment reduced G‐CSF and IL‐6 secretion, improved erythropoiesis, and selectively modulated brain transcriptional changes. Single‐nucleus RNA‐seq projection mapping predicted that the transcripts altered by combined injury were broadly enriched across vascular and glial cell types. Targeted expression profiling identified a shared brain‐liver signature characterized by an IL‐1/6‐dependent inflammatory response alongside an interleukin‐independent stress response. These findings highlight IL‐1‐responsive inflammatory signals and endocrine stress interactions within a multi‐organ network that shapes the early brain transcriptional response to the combined injury.

## Graphical abstract

Concomitant burn injury and blast‐induced mild TBI lead to a magnified neuroimmune response shaped by endocrine stress‐related pathways and IL‐1R1‐dependent inflammatory signals, which also produce cytokine‐mediated impairment of erythropoiesis. These integrated systemic and CNS responses illustrate how inflammation, hematopoietic adaptation, and early brain transcriptional programs interact within a multi‐organ network.

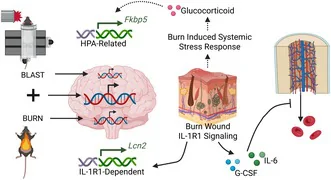

## 1 Introduction

Burn injury to the skin initiates a complex systemic response involving immune, vascular, metabolic, and neuroendocrine alterations that can lead to dysfunction across multiple organ systems [1, 2]. Increasing evidence suggests this response exerts a sustained impact on the central nervous system (CNS), with inflammatory and metabolic processes likely playing a key role in the pathology [3]. Most of the mechanistic insights come from animal studies, primarily rodents, as human data remain limited and are largely derived from postmortem analysis or case reports. Experimental models have demonstrated blood–brain barrier (BBB) disruption and neuroinflammation. In rat models of severe burn injury, involving total body surface area (TBSA) of 40% or more, researchers have observed increased BBB permeability, cerebral edema, elevated proinflammatory cytokines, microglial activation, and apoptosis within the brain [4, 5, 6, 7, 8, 9]. Mouse studies employing moderate burns, 15% TBSA when specified, also report BBB permeability, increased proinflammatory cytokines, apoptosis, and microglial activation [10, 11, 12, 13]. While the brain has not been definitively established as a primary source of proinflammatory cytokine secretion, many prior studies identified TNF‐α, IL‐1β, and IL‐6 as inflammatory markers associated with neuroinflammation and vascular permeability following burn injury, and targeting inflammatory cascades has demonstrated therapeutic effects. Interventions such as anti‐TNFα antibodies or doxycycline have been shown to reduce MMP activity and BBB leakage in rats [7], and therapies such as gelsolin [10], pentoxifylline [8], estradiol [9], and umbilical cord‐derived mesenchymal stem cells [12] appear to mitigate neuropathology through anti‐inflammatory and endothelial‐stabilizing mechanisms.

In recent military conflicts, explosions have been the leading cause of burn injuries [14, 15] and frequently result in complex cases where a blast‐induced traumatic brain injury (TBI) overlaps with a burn injury encompassing less than 20% of TBSA [16]. Blast‐induced TBI may result from a combination of mechanisms, including blast wave overpressure, acceleration‐deceleration forces, and direct head impact or penetration, producing a heterogeneous spectrum of injuries ranging from mild to severe. Human and animal studies suggest underlying pathological features of blast‐induced TBI include vascular disruption, axonal injury, and neuroinflammation, with emerging evidence indicating that even mild blast exposures can have lasting effects on neurological function [17, 18]. Moreover, data from a large national cohort indicate that the presence of co‐occurring TBI across a range of burn severities is associated with significantly increased mortality compared to burns alone or burns that overlap with non‐TBI trauma [19]. The investigators proposed several mechanisms by which the combined injury may worsen outcomes beyond what would be expected from either injury alone. These include conflicting fluid management strategies, in which aggressive burn resuscitation may exacerbate cerebral edema; perioperative hypotension associated with surgical burn wound debridement, which may reduce cerebral perfusion and worsen secondary brain injury; and systemic inflammation triggered by burn injury, which may amplify TBI‐associated neuroinflammatory and vascular responses. Anemia and transfusion thresholds may be another important consideration for patients with overlapping burn and TBI. Greater severity of anemia at admission and during hospitalization independently predicted worse neurologic outcomes following TBI, with each 1 g/dL increase in hemoglobin associated with a 33% higher likelihood of favorable recovery [20], and recent trials suggest TBI outcomes may be improved by maintaining hemoglobin levels at ≥ 9–10 g/dL [21, 22]. Conversely, transfusion guidelines for burn care recommend maintaining a hemoglobin level of ≥ 7 g/dL [23]. Even with this conservative threshold, burn patients often receive an amount of packed red blood cells that is roughly equivalent to their total vascular volume [24], and therapies such as erythropoietin (EPO) [25, 26, 27, 28] and iron [29] supplements do not effectively promote erythropoiesis in burn or trauma patients, consistent with inflammation driven anemia [30, 31]. Taken together, the convergence of overlapping injury mechanisms and competing clinical priorities underscores the need for deeper investigation into the pathophysiology of concomitant burn and TBI.

To investigate the pathophysiological response to concomitant burn and TBI, we examined systemic cytokine secretion, hematopoietic alterations, and transcriptional changes within the brains of mice subjected to a blast‐induced mild TBI and 15% TBSA flame burn for comparison to isolated injuries and control subjects. We also assessed IL‐1‐targeted therapies in the model of combined injury based on our prior work that identified a key role for wound‐associated IL‐1/MyD88‐dependent G‐CSF and IL‐6 secretion in mediating post‐burn anemia [32]. Together, our results suggest that a predominant effect of the combined injury occurs in the brain, where an amplified transcriptional response associated with inflammatory and endocrine stress‐responsive pathways arises across multiple cell types. These changes develop within the context of IL‐1‐dependent systemic inflammation and endocrine stress‐related transcriptional activity, consistent with a multi‐organ network that may influence early brain vulnerability.

## 2 Materials and Methods

### 2.1 Injury Models

Eight‐week‐old male or female C57BL/6J (stock #000664) mice were obtained from Jackson Laboratory (Bar Harbor, ME). Mice were maintained in groups (≤ 5/cage, no single housing) with a 14‐h light:10‐h dark cycle, provided food and water *ad libitum* and allowed to acclimate one week prior to experimentation. The concomitant injury model was developed by combining established models of flame burn injury [33] and mild TBI induced by blast [34]. Briefly, subjects were randomized to the concomitant injury group at the cage level, anesthetized with 4% isoflurane delivered via a precision vaporizer (VetEquip), and placed with heads exposed perpendicular to a shock tube. The burst of a 0.003″ thick Mylar membrane within the shock tube, driven by compressed gas, was used to generate blast waves. Immediately following the blast, mice were covered with a flame‐resistant template exposing an area of shaved dorsal skin encompassing 15% of TBSA. The target area was saturated with 0.5 mL of absolute ethanol, ignited for precisely 10 s to generate a full‐thickness insensate burn lesion, and 1 mL of saline was administered by the intraperitoneal (i.p.) route for volume resuscitation. Subjects designated as TBI or burn alone were manipulated identically to the combined injury group except for alcohol ignition or bursting of the Mylar membrane, respectively. Sham subjects were also manipulated identically to the combined injury group with the exceptions of alcohol ignition or bursting of the Mylar membrane. Mice were sacrificed by i.p. injection of Euthasol (Virbac, Fort Worth, TX). All procedures were approved by the University of Cincinnati Institutional Care and Use Committee.

### 2.2 Righting Reflex Time

Righting reflex time (RRT) was used as a standard behavioral measure of acute blast‐induced TBI severity [35, 36]. Immediately after the injury procedure, mice were placed supine and the time required to regain a prone posture was recorded in seconds.

### 2.3 Analysis of Serum Biomarkers and Circulating Cells

Blood for biomarker measures was collected in serum separator tubes (Microvette 500 Z‐gel, Sarstedt). The isolated serum was stored at −80°C until quantification of G‐CSF and IL‐6 using Milliplex MAP kits (Millipore, Billerica, MA), corticosterone by ELISA (Enzo Life Sciences, catalog no. ADI‐900‐097), or Lipocalin‐2 (LCN2) by Quantikine ELISA (R&D Systems, catalog no. MLCN20). Blood for cell counts was collected in EDTA‐coated tubes (Microvette 500 K3E, Sarstedt), and cell counts were performed on an ADVIA 2120i Hematology System (Siemens Healthcare).

### 2.4 Bone Marrow Cell Analysis

Bone marrow cells were isolated by flushing each femur with 1.5 mL of ice‐cold HBSS and passed through a 70 μm filter. Isolated cells were spun at 400× *g* in a 4°C centrifuge, resuspended in DPBS containing 1/1000 dilution of the viability dye eFluor 780 (eBioscience) and incubated at 4°C for 30 min according to manufacturer's instructions. After washing two times in ice‐cold FACS buffer (DBPS containing 0.5% BSA and 0.1% sodium azide), cells were blocked with rat serum and Mouse Fc Block (BD Biosciences) for 10 min on ice before staining with fluorescent labeled antibodies, TER‐119 (clone TER‐119, FITC labeled) and CD45 (clone 30‐F11, BV421 labeled), for 30 min. Cells were washed three times in ice‐cold FACS buffer before running on an Attune (Thermo Fisher) flow cytometer and data were analyzed with FCS Express 7 (Dotmatics).

### 2.5 Cytokine Blockade

Prophylactic IL‐1α and IL‐1β blockade was achieved by i.p. injection of a 100 μL PBS solution containing 200 μg of anti‐IL‐1α (clone ALF‐161, Bio X Cell, catalog no. BE0243) and 200 μg of anti‐IL‐1β (clone B122, Bio X Cell, catalog no. BE0246) administered as a cocktail. Control animals received 400 μg of Armenian hamster IgG isotype control (Bio X Cell, catalog no. BE0091) in the same volume. For prophylactic administration, injections were given 12 h prior to injury and again immediately after injury as in our prior study [32]. For therapeutic administration, a single i.p. injection of the anti‐IL‐1α/β cocktail or isotype control was administered immediately post‐injury. For therapeutic comparison of IL‐1α/β blockade and IL‐6 blockade, all mice received an equivalent 600 μg total dose of antibodies diluted in PBS via a single 200 μL i.p. injection immediately following injury. The isotype control groups received 200 μg of rat IgG1 (clone TNP6A7, Bio X Cell, catalog no. BE0290) combined with 400 μg of the Armenian hamster IgG isotype control. The IL‐6 blockade group received 200 μg of rat anti‐mouse IL‐6 (clone MP5‐20F3, Bio X Cell, catalog no. BE0046) combined with 400 μg of the Armenian hamster IgG isotype control. The IL‐1α/β blockade group received 200 μg of the anti‐IL‐1α and 200 μg of the anti‐IL‐1β combined with 200 μg of the rat IgG1 isotype control. For each regimen, blood and tissues were collected 6 h after injury for endpoint measures.

### 2.6 IL‐1R1 Antagonism

Anakinra (Kineret, Sobi) was diluted in saline to deliver 150 mg/kg in a 100 μL volume via i.p. injection. For 6‐h endpoints, a single dose of anakinra or saline (vehicle control) was administered immediately after injury. For examination of hematological changes 7 days after injury, injections were given immediately post‐injury, again at 8 h, and then every 12 h through the day prior to tissue collection.

### 2.7 Brain RT‐PCR

Ipsilateral half brain or whole brain RNA was isolated with RNAzol RT (Molecular Research Center, catalog no. RN190) and converted to cDNA using the High‐Capacity cDNA Reverse Transcription Kit (Applied Biosystems, Foster City, CA). Power SYBR Green PCR Master Mix (Applied Biosystems, Foster City, CA) was used to amplify DNA fragments through 40 cycles at 95°C for 15 s followed by 60°C for 1 min. Primer sequences are provided in Table S1. Gene expression was normalized to *Actb*.

### 2.8 Bulk RNA‐Seq

PolyA RNA‐seq was performed by the Genomics, Epigenomics and Sequencing Core at the University of Cincinnati. Briefly, mirVana kits (Thermo Fisher) were used to isolate total RNA from ipsilateral half brains (multiple‐injury studies) or whole brains (anakinra studies), and the quality of total RNA was confirmed on a bioanalyzer (Agilent). To enrich polyA RNA for library preparation, NEBNext Poly(A) mRNA Magnetic Isolation Module (New England BioLabs) was used with 1 μg (multiple‐injury studies)/500 ng (anakinra studies) of good quality total RNA as input. NEBNext Ultra II Directional RNA Library Prep kits (New England BioLabs) were used for unique dual index library preparation under PCR cycle number of 8 (multiple‐injury studies)/9 (anakinra studies). After library QC and Qubit quantification (Thermo Fisher), the normalized libraries were sequenced using NextSeq 2000 Sequencer (Illumina). Under the setting of PE 2 × 61 bp, ~30 M read pairs were generated for each sample. Sequenced reads were demultiplexed in Illumina BaseSpace Sequence Hub using default settings, which also produced adapter‐trimmed FASTQ files. Trimmed reads were processed in BaseSpace with the RNA‐Seq Alignment app (v 2.0.2), where alignments to the *Mus musculus* reference genome (UCSC mm10) were performed by STAR, and transcript abundances were quantified by Salmon within the RNA‐Seq Differential Expression app (v 1.0.1). The estimated read counts (“NumReads” column) from each Salmon output were merged into a count matrix for analysis in Partek Flow (Illumina). Differential expression analyses were performed with DESeq2 using models that included design formulas to account for gender and batch (detailed in Supplemental RNA‐seq QC Analysis, Text S1). Sample metadata, input values, DESeq2 results, and corrected feature counts used for visualizations are provided for multiple‐injury studies (Data S1) and anakinra studies (Data S2).

**FIGURE 1:**
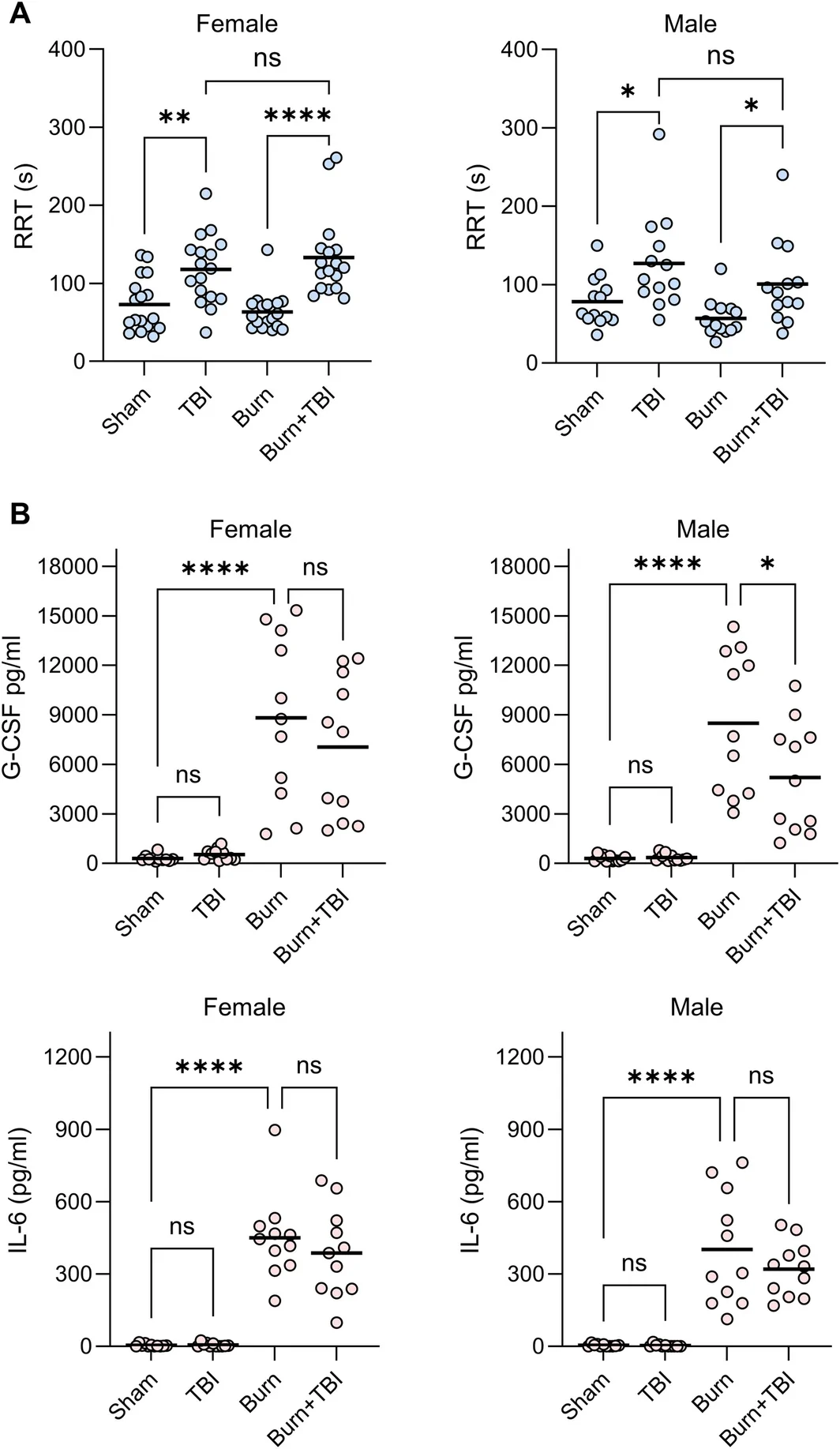
Mild TBI increases righting reflex time while burn injury drives systemic G‐CSF and IL‐6 secretion in the combined injury model. Righting reflex times (RRT) were recorded in seconds immediately post‐injury as a measure of acute TBI severity. (A) Pooled RRT data from three independent experiments are shown with a line at the mean for female (*n* = 17/group) and male (*n* = 13/group) mice. (B) Serum was isolated at 24 h post‐injury from separate cohorts of female and male mice for quantification of G‐CSF and IL‐6 using milliplex bead assays. Cytokine data represent pooled results from two independent experiments, plotted with a line at the mean for female (*n* = 11/group) and male (*n* = 11/group). An ANOVA with a post hoc Holm‐Sidak test was used for comparisons shown with lines. *p < 0.05, **p < 0.01, ****p < 0.0001, ns, not significant.

**FIGURE 2:**
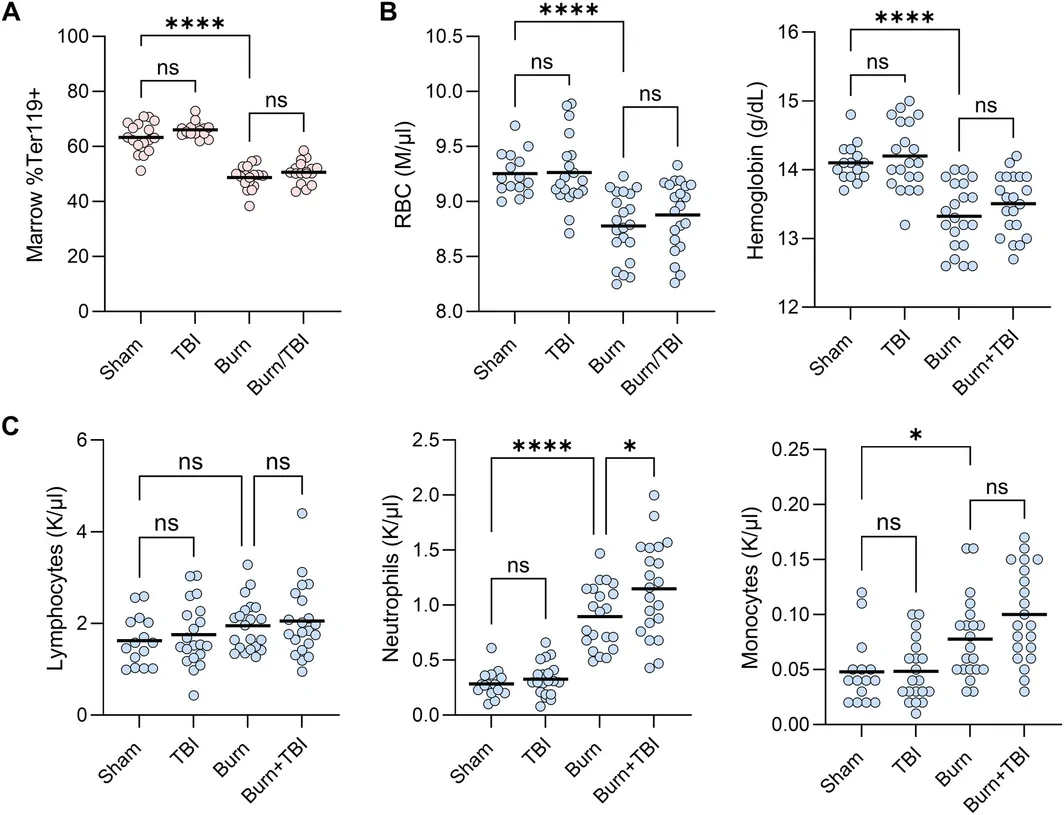
Hematopoietic changes in the combined injury model are primarily driven by burn injury. Bone marrow was isolated from female mice on day 7 post‐injury to assess erythroid (Ter119+) composition by flow cytometry. (A) Pooled data from three independent experiments are shown with a line at the mean (*n* = 16–17/group). Whole blood was collected from female mice on day 7 post‐injury to quantify (B) red blood cell counts, hemoglobin concentrations, and (C) white blood cell counts using an ADVIA 2120i Hematology System. Blood measurements represent pooled results from three independent experiments (*n* = 15–21/group), plotted with a line at the mean. An ANOVA with a post hoc Holm‐Sidak test was used for comparisons shown with lines. *p < 0.05, ****p < 0.0001, ns, not significant.

### 2.9 GSEA Analysis

Gene set enrichment analysis was performed using the GSEA desktop application (version 4.4.0, Broad Institute). Normalized gene counts from bulk RNA‐sequencing (multiple‐injury studies) were used as input, and the analysis compared the burn/TBI group to all other groups combined (sham, TBI, and burn). Gene set permutations (*n* = 5000) were used to estimate statistical significance. This approach was chosen to accommodate the multi‐group experimental design and to ensure valid inference when comparing one condition against a composite baseline. Brain‐related gene sets were obtained from the Brain.GMT database, a curated collection of gene sets relevant to nervous system function, brain cell types, and neuropsychiatric disorders [37]. The mouse‐specific version (BrainGMTv2_MouseOrthologs.gmt.txt) was used to ensure compatibility with the expression dataset. In addition, two custom files (.gmt) were constructed from differentially expressed gene lists derived from the brain transcriptomic data of mice treated with a high‐affinity monoclonal antibody targeting corticotropin‐releasing factor (CRF). The study demonstrated CRF blockade suppressed hypothalamic–pituitary–adrenal (HPA) axis activity and identified genes upregulated or downregulated in the brain using an adjusted *p* value of < 0.05 without considering a fold change [38].

### 2.10 Single‐Nucleus RNA‐Seq

Whole brains were placed into an acrylic mouse brain matrix (Ted Pella, catalog no. 15051) to ensure consistent anatomical orientation. A 1‐mm sagittal section was obtained from the ipsilateral (left) hemisphere, immediately lateral to the midline, using the matrix to guide sectioning. This region was collected from three mice: one sham control and two subjected to combined burn and blast‐induced mild TBI at 6‐ or 24‐h post‐injury. Each section was flash‐frozen and stored in liquid nitrogen until nuclei were isolated using the Chromium Nuclei Isolation Kit with RNase Inhibitor (10× Genomics, product code 1000494), following the manufacturer's recommendations. The protocol included an additional wash to reduce myelin debris and passage through a 40 μm FlowMi cell strainer to minimize aggregates. The percentage of intact nuclei was 86% for the sham control, 81% for the 6‐h sample, and 83% for the 24‐h sample. Approximately 9600 nuclei were loaded per sample for gel bead‐in‐emulsion (GEM) generation using the Chromium 10× Genomics 3′ v3.1 platform. Single‐nucleus libraries were prepared following the 10× Genomics protocol, including 14 cycles of cDNA amplification, and sequenced on an Illumina NovaSeq 6000. Resulting fastqs were processed through the CellRanger pipeline v6.1.2 using 10× Genomics mm10‐2020‐A reference genome with the setting “include introns” set to true. Using the filtered_feature_bc_matrix as input, doublets were minimized using DoubletFinder [39]. To annotate cell clusters, we integrated single‐nucleus RNA‐seq data from sham, 6‐h, and 24‐h post‐injury samples and performed unsupervised clustering using the R v4.4 library Seurat v4.4 [40]. Nuclei expressing > 500 genes, < 5% mitochondrial reads, and > 1000 RNA counts were retained for analysis. Each sample was normalized by SCTransform v2, using the glmGamPoi method and the number of RNA molecules per nuclei and cell cycle effect were regressed out. Samples were integrated with common anchor genes using the Canonical Correlation Analysis method to minimize sample to sample variation. Cell clusters were determined by the Louvain algorithm using a resolution of 0.2. UMAP dimension reduction was done using the first eighteen principal components. Marker genes for each cell type were calculated using the Wilcoxon Rank Sum test returning only genes that are present in a minimum of 25% of the analyzed cluster and have a minimum differential cell expression of 30%. Cell‐type identities were assigned based on canonical markers and the output of Seurat's FindAllMarkers function, which generated a custom marker gene list for each cluster. Integration across conditions improved resolution of underrepresented populations. Subclustering of populations was examined using Loupe Browser (10× Genomics) to refine cell‐type annotations and we observed the Loupe t‐SNE projection yielded more coherent spatial grouping of transcriptionally similar clusters compared to the default UMAP in Seurat. Therefore, we exported the Loupe t‐SNE coordinates and reimported them into Seurat for downstream visualization. This adjustment improved interpretability of spatial relationships without altering cluster membership. Separately, to infer the likely cellular origins of transcriptional changes identified in bulk RNA‐seq, we used the tissue_scMappR_custom function from the scMappR package [41]. DEGs from the 6‐h burn/TBI vs. sham bulk RNA‐seq comparison were used as input, and enrichment was assessed against the cluster‐specific marker gene list derived from the integrated snRNA‐seq dataset. This analysis predicts the cell types with enriched expression of each DEG based on injury‐agnostic transcriptional identity and does not model changes in cell‐type abundance or activation state.

### 2.11 Immunofluorescence Staining

For whole‐brain and liver staining, mice were transcardially perfused with ice‐cold PBS followed by ice‐cold 4% paraformaldehyde (PFA). Excised tissues were post‐fixed in 4% PFA overnight and cryoprotected in 30% sucrose in PBS at 4°C. Brain and liver sections (20 μm) were obtained using a cryostat and slide‐mounted for immunostaining. Sections were blocked with 5% donkey serum and 0.3% Triton X‐100 in PBS for 1 h at room temperature, then incubated overnight at 4°C with goat anti‐mouse LCN2 polyclonal IgG (1:50; R&D Systems, catalog no. AF1857) and DyLight 488‐conjugated Tomato Lectin (1:200; Vector Laboratories, catalog no. DL‐1174‐1). Sections were incubated with an Alexa Fluor 647‐conjugated donkey anti‐goat IgG secondary antibody (1:500; Jackson ImmunoResearch, catalog no. 705‐605‐003) for 1 h at room temperature to visualize LCN2 and coverslipped using VECTASHIELD mounting medium with DAPI (Vector Laboratories). For staining of isolated brain vasculature, flash‐frozen whole‐brain tissue was homogenized in ice‐cold buffer (1M sucrose, 1% BSA, DEPC‐treated water) using gentleMACS C Tubes and program Lung 02.01 as previously described [42]. The homogenate was mixed by gentle inversion before centrifugation at 3200× *g* for 30 min at 4°C. The pellet was resuspended in 1 mL of ice‐cold PBS, transferred to chamber slides (50 μL/chamber) and left open‐faced in a 37°C incubator to dry overnight. The next day, vessels were fixed with 4% PFA for 10 min, washed with PBS, and then blocked and stained similar to tissue sections. All Fluorescence images were acquired using a Leica Stellaris 8 Confocal Microscope.

### 2.12 Statistics

Statistical analysis was performed using GraphPad Prism 10. An ANOVA with post hoc Holm‐Sidak's test was used for comparisons involving three or more groups as specified in the figure legends. Significant differences were determined at *p* values of < 0.05. Statistical thresholds used for bulk RNA‐seq are described within the results section.

## 3 Results

### 3.1 Burn Injury Drives Systemic Cytokine Secretion and Hematopoietic Changes in the Combined Injury Model

Righting reflex time (RRT) was assessed as an acute metric of blast‐induced TBI severity and showed a prolongation in mice exposed to TBI, consistent with prior reports for the model of blast‐induced mild TBI [35, 36], whereas burn injury did not alter the response (Figure 1A). Burn injuries induce systemic secretion of inflammatory mediators, including TNFα, IL‐1β, IL‐6, and G‐CSF which are elevated in rodent models of burn injury [43] and human burn victims [44, 45]. Except for G‐CSF, these cytokines have been used as markers of post‐burn neuroinflammation [4, 8, 9, 10, 12, 13]; however, a brain source was commonly inferred from homogenized tissue protein measures, and mRNA‐based studies acknowledge infiltrating or residual circulating immune cells as a potential source. Wound‐based IL‐1/MyD88 signaling plays a key role in driving systemic G‐CSF and IL‐6 secretion in our burn model [32], altering hematopoiesis and iron utilization in a manner that promotes anemia. Given these cytokines impair erythropoiesis following burn injury [32, 33, 46, 47, 48] and the potential link between low hemoglobin and poor neurological outcomes [20, 21, 22], we assessed circulating G‐CSF and IL‐6 levels as measures of systemic inflammation along with associated changes in hematopoietic output across injury models. Quantification of circulating G‐CSF and IL‐6 on post‐injury day one indicated the burn induced secretion of these cytokines was not augmented by combined injury and there was no evidence that the mild TBI alone induced their secretion (Figure 1B). Hematopoietic alterations were examined in female mice on post‐injury day seven by assessing erythroid composition of the bone marrow using flow cytometry, and blood cells were quantified on an automated hematology analyzer. Combined injury did not amplify a burn induced reduction in erythroid composition of the bone marrow, circulating red blood cell counts, or hemoglobin levels, and there was no evidence that mild TBI alone elicited changes in these parameters (Figure 2A,B). Combined injury led to a statistically significant increase of neutrophils compared to burn alone, but there was no evidence that the mild TBI alone altered white blood cell counts (Figure 2C). Hematological assessment was performed in one cohort of male mice and reflected similar burn‐induced changes (Figure S1). Together, these data identified burn injury as the predominant driver of systemic inflammation and hematopoietic alterations in the model of combined injury.

### 3.2 Combined Injury Amplifies the Transcriptional Response in the Brain Compared to Isolated Injuries

Although burn injury appeared to drive the systemic inflammation and hematopoietic changes in the combined injury model, the brain's response could still be shaped by both the local effects of mild TBI and the peripheral inflammation triggered by burn. To assess this, we performed bulk RNA‐seq on the ipsilateral half‐brain harvested at six hours post‐injury to determine whether combined injury elicits a different pattern of gene expression changes compared to either injury alone. Differential expression analysis comparing each injury group to sham (*Q* ≤ 0.01; fold‐change ≥ ±1.5) revealed a markedly more robust transcriptional response in the combined injury group and that burn injury alone elicited a more robust transcriptional response than mild TBI (Figure 3A,B). A small set of genes met our significance thresholds in the mild TBI group, including *Mmp19* and *Timp1*, which were upregulated in both TBI and combined injury but not burn alone, and *Slc22a6*, which was downregulated in these same groups. Other transcripts, such as *Lrg1* and *Pla2g3*, showed a graded increase from TBI to burn to burn/TBI. Together, these patterns suggest that the amplified transcriptional response to combined injury incorporates elements of both injury models. We also conducted differential expression analysis comparing the combined injury group to each of the single injury models (*Q* ≤ 0.01; fold‐change ≥ ±1.5), identifying unique genes as those that met our significance thresholds in every pairwise comparison. This analysis identified 24 significantly upregulated genes and 9 significantly downregulated genes (Figure 3C). Among the upregulated genes, *Txnip* (thioredoxin‐interacting protein) has been linked to mitochondrial dysfunction and neuronal apoptosis [49], while *Ddit4* (REDD1) has been reported to promote neuronal death and inflammation [50]. *Pmaip1* (NOXA) can induce mitochondrial permeabilization and caspase activation [51], and *Tlr7* (Toll‐like receptor 7) has been reported to promote inflammation and caspase‐3‐dependent apoptosis [52, 53]. Conversely, several upregulated genes have been reported to play anti‐inflammatory roles. *Ackr2* (atypical chemokine receptor 2) has been recognized as a negative regulator of neuroinflammation, limiting chemokine availability and immune cell recruitment in the CNS [54]. *Nfkbia* (IκBα) is a key inhibitor of NF‐κB that has been shown to restrain pro‐inflammatory transcriptional programs after brain injury [55]. *Zfp36* (tristetraprolin) is an RNA‐binding protein that has been reported to promote degradation of mRNAs encoding inflammatory cytokines, acting as part of a negative feedback loop to terminate excessive inflammatory signaling [56]. Downregulated genes included two factors that play a role in angiogenesis. *Fzd10* (frizzled‐10) serves as a Wnt receptor that is important for CNS angiogenesis and neurovascular development [57], while *Kdr* (Vegfr2) is a receptor for VEGF‐A and is essential for endothelial survival, proliferation, and vascular regeneration following CNS injury [58]. Together, these findings suggest that, beyond the magnified or shared responses to single injuries, combined burn and mild TBI is associated with a distinct set of transcriptional changes, involving genes that have previously been linked to pro‐apoptotic/stress signaling, modulation of inflammation, and angiogenesis.

**FIGURE 3:**
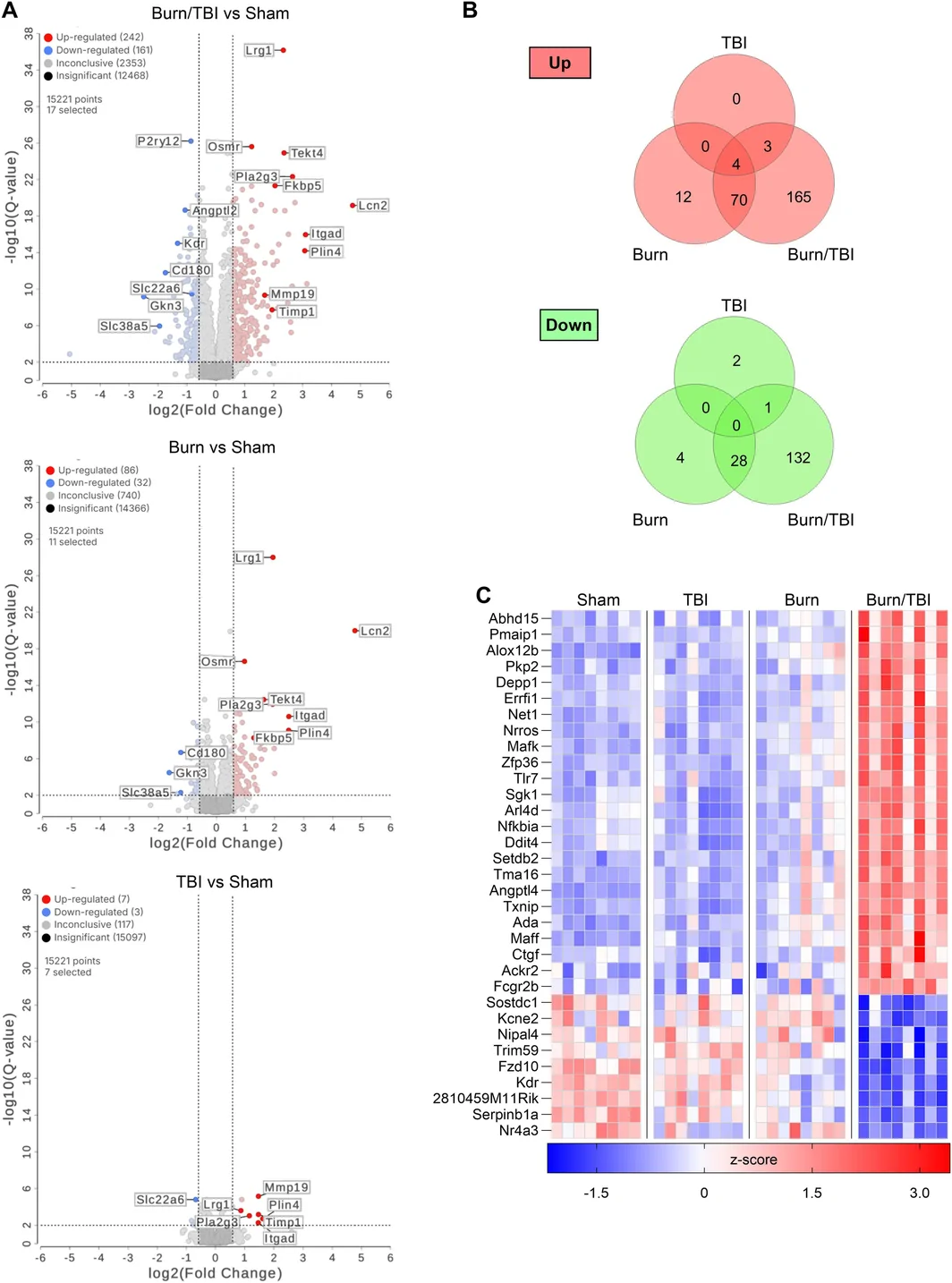
Combined injury amplifies molecular changes in the brain. Ipsilateral half‐brain tissue was isolated from cohorts of male and female mice at 6 h post‐injury for RNA‐seq (*n* = 4 per sex per group). (A) Volcano plots showing differential expression relative to sham, with dashed lines representing significance thresholds (*Q* ≤ 0.01 and fold change ±1.5). (B) Venn diagrams illustrating the number of genes upregulated or downregulated in each injury condition compared to sham controls. (C) Heat map of genes uniquely modified by combined injury, defined as those meeting significance thresholds (*Q* ≤ 0.01 and fold change ≥ ±1.5) in all pairwise comparisons between combined injury and each single‐injury model.

### 3.3 Inflammatory and Glucocorticoid Signaling Responsive Genes Are Enriched in the Early Transcriptional Response to Combined Injury

To further interrogate the transcriptional changes induced by combined burn and mild TBI, we performed gene set enrichment analysis (FDR *Q* < 0.05; combined injury versus rest, where rest = sham, burn alone, and mild TBI alone pooled) using the Brain.GMT database, a curated collection of brain‐relevant pathways, cell‐type signatures, neuropsychiatric gene sets [37]. We also incorporated two custom gene set files, ANTI_CRF_UP and ANTI_CRF_DOWN, that we derived from a prior study in which a high‐affinity monoclonal antibody was used to neutralize CRF, thereby suppressing HPA‐axis activity and broadly altering glucocorticoid‐responsive genes in the brain [38]. Here, ANTI_CRF_UP denotes genes upregulated by CRF blockade/HPA suppression, whereas ANTI_CRF_DOWN denotes genes downregulated by CRF blockade/HPA suppression. Inclusion of these CRF‐blockade signatures was motivated by consistent findings of sustained cortisol elevation in humans [59, 60, 61] and corticosterone elevation in mice [62, 63] after burn injury, reflecting activation of the HPA axis and its impact on immune, metabolic, and neural processes. This dual‐pronged approach allowed us to assess both general brain‐focused pathways and a specific stress response relevant to burn‐associated neuroendocrine activation. Among the significant gene sets, we observed positive enrichment of glucocorticoid and inflammatory programs and negative enrichment of multiple vascular/endothelial signatures (Data S3). To prioritize signatures, we ranked all sets by normalized enrichment score (NES) and plotted the top three positively and negatively enriched sets (Figure 4A). In this snapshot, ANTI_CRF_UP was negatively enriched while ANTI_CRF_DOWN was positively enriched, indicating that combined injury drove transcriptional shifts opposite to CRF blockade/HPA suppression. By contrast, gene sets from dexamethasone and LPS studies showed concordant directionality, with up signatures positively enriched and down signatures negatively enriched, consistent with glucocorticoid and inflammatory programs being engaged by combined injury. We next generated heat maps of the ANTI_CRF_UP and ANTI_CRF_DOWN leading‐edge genes, which revealed a graded shift from TBI to burn to combined burn/TBI relative to sham controls (Figure 4B). Among the leading‐edge genes, *Nr3c1* was downregulated by combined injury, aligning with prior evidence that ligand binding during heightened HPA axis activity can directly downregulated glucocorticoid receptor expression [64]. These transcriptional changes were concordant with an increased level of corticosterone in mice subjected to combined injury (Figure 4C). Finally, we displayed the ANTI_CRF_UP and ANTI_CRF_DOWN gene lists in volcano plots to assess how many DEGs in our burn/TBI versus sham comparison could be related to HPA axis activity (Figure 4D). Nearly all ANTI_CRF_UP genes (genes suppressed by HPA activity) were downregulated, with 63 meeting the significance criteria we applied, whereas nearly all ANTI_CRF_DOWN genes (genes increased by HPA activity) were upregulated, with 87 meeting the significance criteria we applied. Given our prior comparison of burn/TBI versus sham revealed 242 upregulated genes and 161 downregulated genes (Figure 3A), these results suggested HPA axis activity could account for more than one‐third of the DEGs we identified as altered by combined injury.

**FIGURE 4:**
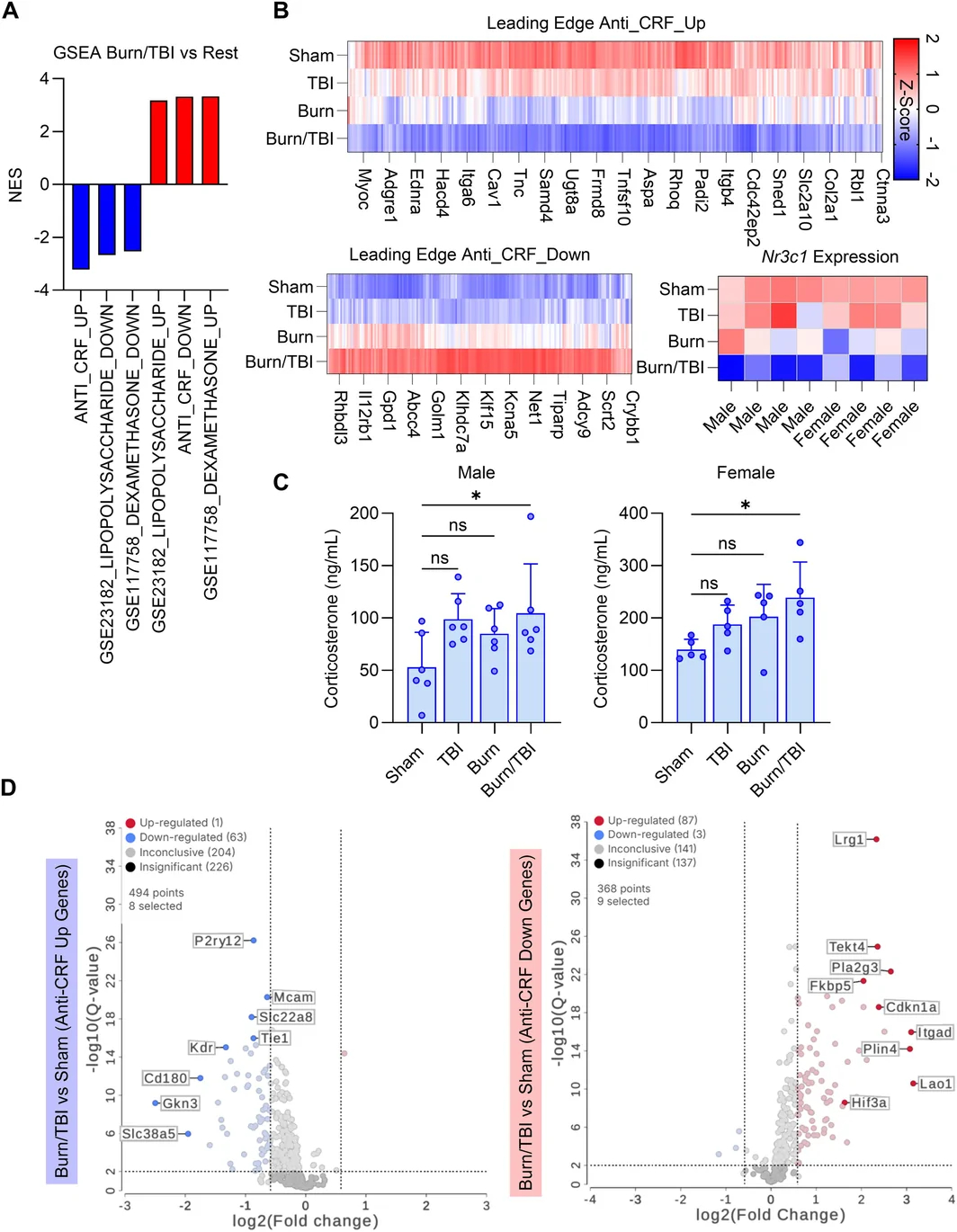
Inflammatory and HPA‐axis signatures are enriched in the brain following combined injury. Gene set enrichment analysis (GSEA) was performed on burn/TBI versus rest (sham, burn, and TBI pooled) incorporating brain‐focused and CRF‐blockade (HPA‐suppression) gene sets. ANTI_CRF_UP contains genes normally downregulated by HPA‐axis activity, whereas ANTI_CRF_DOWN contains genes normally upregulated by HPA‐axis activity, together providing inverse signatures of HPA‐axis engagement. (A) Top three positively and negatively enriched signatures based on normalized enrichment score (NES). (B) Heat maps of ANTI_CRF_UP and ANTI_CRF_DOWN leading‐edge genes across groups and *Nr3c1* by group for each sample. (C) Serum corticosterone levels for male (*n* = 6/group) and female (*n* = 5/group) mice at 6 h post‐injury. (D) Volcano plots of burn/TBI versus sham that only display genes included in the ANTI_CRF_UP (left) and ANTI_CRF_DOWN (right) gene sets. An ANOVA with a post hoc Holm‐Sidak test was used for comparisons shown with lines. * *p* < 0.05, ns, not signficant.

### 3.4 IL‐1α/β Neutralization Attenuates Systemic Cytokine Secretion and Brain Expression of Inflammatory Markers Following Combined Burn/TBI

IL‐1α and/or IL‐1β binding to IL‐1R1 recruits MyD88 to TIR domains of IL‐1R1 and its co‐receptor IL‐1R3, leading to activation of IRAK1/4 and downstream production of inflammatory cytokines [65]. Because our prior work established a central role for IL‐1/MyD88 signaling in the post‐burn inflammatory response [32], we applied the same prophylactic IL‐1α/β neutralization protocol to test whether blocking IL‐1 signaling attenuates G‐CSF and IL‐6 secretion in the combined injury model. We also examined whether this approach reduced *Lcn2* (lipocalin‐2) and *Lrg1* (leucine‐rich α‐2 glycoprotein 1) expression in ipsilateral brain tissue, as these genes were strongly upregulated in our bulk RNA‐seq data (Figure 3A) and are widely recognized markers of inflammatory activation in the brain and other tissues [66, 67]. Prophylactic administration of IL‐1α/β neutralizing antibodies 12 h before injury and again immediately post‐injury significantly reduced cytokine secretion and inflammatory gene expression at 6 h post‐injury, although *Lrg1* attenuation was less pronounced than *Lcn2* attenuation (Figure 5A). A single therapeutic dose of IL‐1α/β neutralizing antibodies immediately following injury also effectively attenuated cytokine secretion and inflammatory marker gene induction at 6 h post‐injury (Figure 5B), suggesting that IL‐1‐targeted therapies could be clinically feasible for mitigating neuroinflammation after concomitant burn and blast‐induced mild TBI. Notably, IL‐1α/β neutralization consistently produced weaker effects on *Lrg1* compared to *Lcn2*, and the magnitude of *Lrg1* attenuation was similar in both prophylactic and therapeutic administration scenarios, suggesting residual *Lrg1* expression was not due to incomplete IL‐1 blockade. Our previous findings further support this interpretation as *Lrg1*, but not *Lcn2*, appeared responsive to HPA‐axis activation (Figure 4D), and IL‐1R1 deficiency in a model of chronic stress has been shown to broadly suppress *Lcn2* but only regionally suppress *Lrg1* expression in the brain [68]. Together, this suggests that *Lrg1* induction in the model of combined injury may be regulated not only by IL‐1R1 signaling but also through additional stress‐responsive pathways.

**FIGURE 5:**
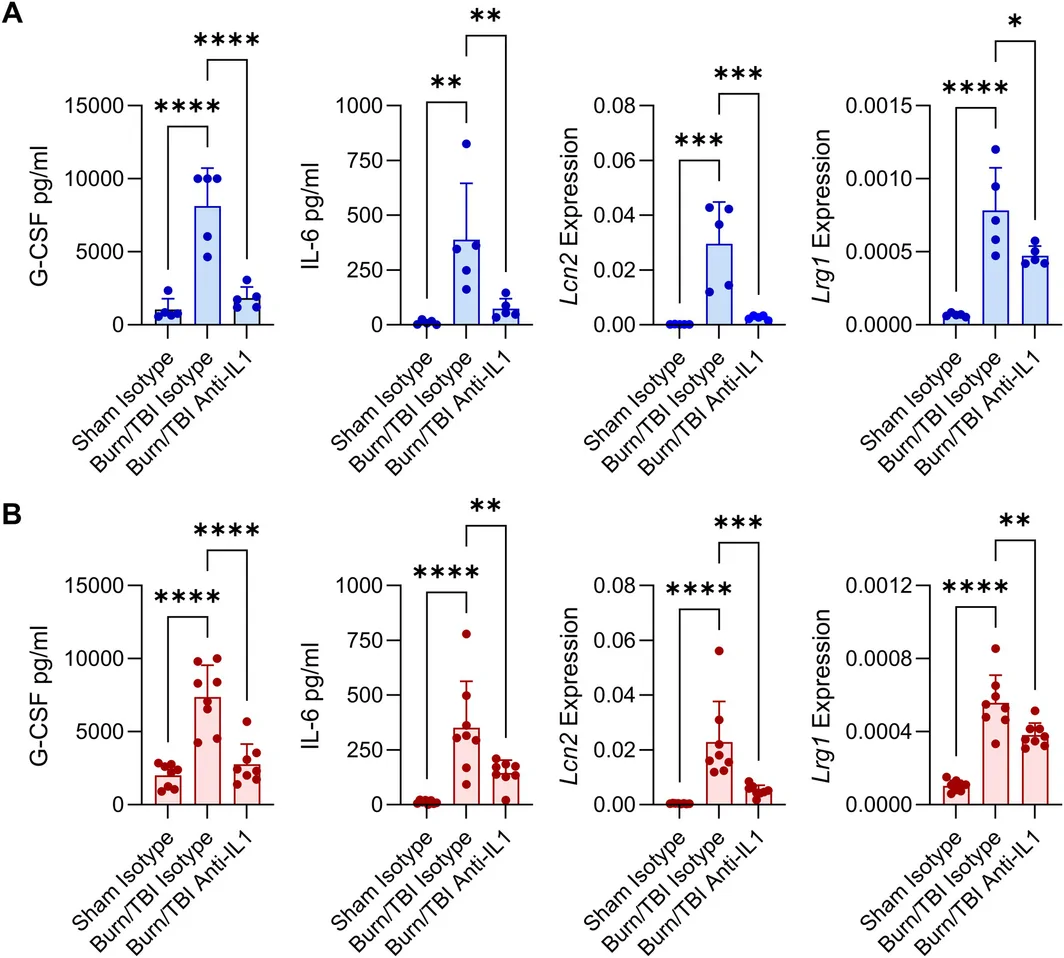
IL‐1α/β blockade attenuates systemic cytokine secretion and inflammatory marker gene expression in the brain following combined injury. Female mice received IL‐1α/β neutralizing or isotype control antibodies in prophylactic or therapeutic regimens. At 6 h post‐injury, serum was collected for quantification of G‐CSF and IL‐6, and the ipsilateral brain hemisphere was harvested for RT‐PCR quantification of *Lcn2* and *Lrg1* as inflammatory marker genes. Cytokine and gene expression results are shown for (A) prophylactic (*n* = 5/group) and (B) therapeutic administration (*n* = 8/group), plotted as mean ± SD. ANOVA with a post hoc Holm‐Sidak test was used for comparisons shown with lines. *p < 0.05, **p < 0.01, ***p < 0.001, ****p < 0.0001.

### 3.5 Therapeutic Administration of Anakinra Over One Week Following Combined Injury Mitigates Hematopoietic Changes That Lead to Anemia Following Combined Injury

Given that IL‐1α/β neutralization effectively reduced systemic cytokine secretion and upregulation of inflammatory marker genes in the brain following combined injury, we next assessed anakinra (recombinant interleukin‐1 receptor antagonist) as a more translational strategy. A prior study reported that anakinra at 3 mg per mouse (~150 mg/kg for a typical 20 g mouse), administered in a repeated‐dosing regimen, effectively blocked G‐CSF‐dependent hematopoietic changes in the bone marrow of mice subjected to polytrauma [69]. Consistent with this, our pilot assessments indicated that both 100 and 200 mg/kg doses administered immediately after combined injury produced similar reductions in G‐CSF & IL‐6 secretion at 6 h post‐injury. On this basis, we selected 150 mg/kg as an effective therapeutic dose for subsequent experiments. To determine whether therapeutic administration of anakinra could attenuate hematopoietic changes in the combined injury model, we administered anakinra immediately after injury, again at 8 h, and then every 12 h until the day prior to harvesting bone marrow cells for flow cytometry analysis and blood for analysis using an automated hematology analyzer. Like IL‐1α/β blockade in our prior study involving burn alone [32], anakinra attenuated the reduction of erythroid cells in the bone marrow and improved circulating red blood cell parameters (Figure 6A,B). Anakinra also attenuated increases of circulating white blood cells following combined injury, with lymphocytes and neutrophils significantly reduced (Figure 6C). Together, these results show that anakinra can mitigate combined injury‐induced hematopoietic alterations in the bone marrow and circulation.

**FIGURE 6:**
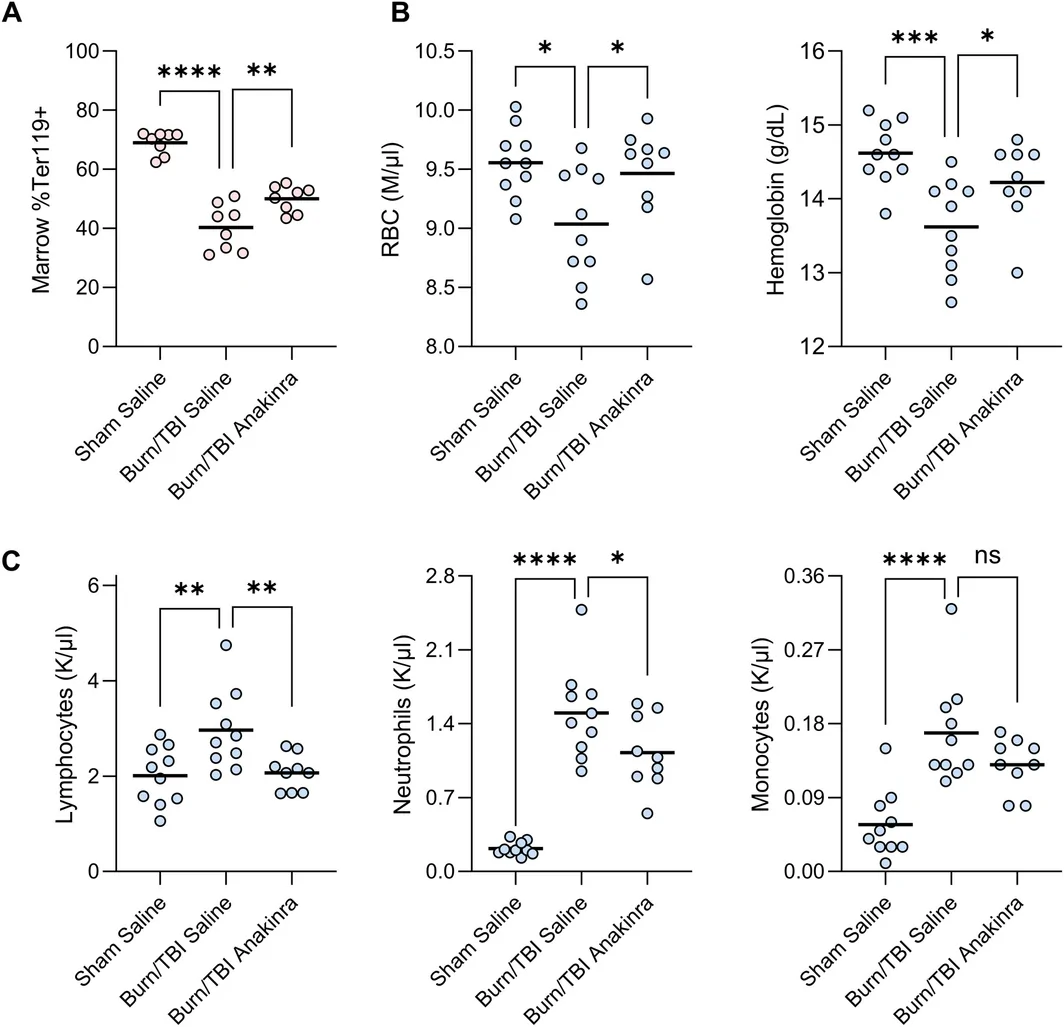
Anakinra attenuates hematopoietic changes induced by combined injury. Female mice were administered Anakinra through day 6 post injury. Bone marrow and peripheral blood were collected on post‐injury day 7 for hematological analysis. (A) Bone marrow erythroid composition determined by flow cytometry is shown with a line at the mean (*n* = 8/group). Whole blood was analyzed using an ADVIA 2120i Hematology System to measure (B) red blood cell counts, hemoglobin concentrations, and (C) white blood cell counts. Blood results are shown with a line at the mean (*n* = 9–10/group). An ANOVA with a post hoc Holm‐Sidak test was used for comparisons shown with lines. *p < 0.05, **p < 0.01, ***p < 0.001, ****p < 0.0001, ns not significant.

### 3.6 Anakinra Attenuates Systemic Cytokine Secretion and Selectively Reshapes Transcriptional Responses Following Combined Injury

To determine if anakinra treatment could mitigate transcriptional changes induced by overlapping burn and blast‐induced mild TBI, we administered 150 mg/kg of Anakinra immediately after injury and 6 h later blood and whole brain tissue were collected for cytokine quantification and RNA‐seq, respectively. Cytokine quantification confirmed anakinra markedly attenuated G‐CSF and IL‐6 secretion following combined injury (Figure 7A). Consistent with our IL‐1α/β neutralization studies, which showed weaker suppression of *Lrg1* compared to *Lcn2*, analysis of normalized counts from whole brain RNA‐seq revealed that anakinra strongly suppressed *Lcn2* expression, while the reduction in *Lrg1* was more modest (Figure 7B). Differential expression analysis of combined injury groups versus sham (*Q* ≤ 0.01; fold‐change ≥ ±1.5) revealed a comparable number of upregulated and downregulated DEGs (Figure 7C), indicating that anakinra did not broadly normalize injury‐induced transcription but instead exerted modest and/or selective effects. In particular, transcripts identified in our prior enrichment analysis as HPA‐axis‐related (e.g., *Fkbp5, Cdkn1a, Plin4, Hif3a, Pla2g3*) remained largely unaffected, while immune‐related transcripts (e.g., *Lcn2, Lrg1, Cd14, Csf2rb*) were modulated by anakinra. Because anakinra produced modest but biologically meaningful modulation of immune and inflammatory transcripts, including *Lcn2* and *Lrg1*, both validated as IL‐1‐dependent in our antibody neutralization studies using RT‐PCR, we applied complementary screening criteria (*Q* ≤ 0.15; fold‐change ≥ ±1.25, requiring a gene to change with injury and again with treatment) to increase sensitivity for genes modulated by both injury and treatment. Using this approach, we first identified transcripts altered by injury (burn+TBI saline vs. sham saline) and then determined which were directionally reversed or augmented by anakinra (burn+TBI anakinra vs. burn+TBI saline). In addition to *Lcn2* and *Lrg1*, anakinra treatment attenuated a broader set of inflammatory genes (Figure 8A). The attenuated profile included chemokines (*Ccl19, Ccl21b*) that have been associated with chronic neuroinflammation [70], innate immune receptors (*Cd14, C5ar1, Csf2rb*), and S100 family DAMPs (*S100a8, S100a9*), prototypical alarmins that have been associated with CNS inflammation [71]. We also observed attenuation of transcriptional control genes (*Runx1, Cebpd, Bcl3*), each previously implicated in inflammatory programs following stroke [72]. Beyond these immune and regulatory transcripts, anakinra reduced induction of *Ch25h*, whose oxysterol product 25‐hydroxycholesterol has been reported to amplify IL‐1β–driven microglial inflammation and tau‐mediated neurodegeneration [73], and is strongly inducible in CNS endothelial cells where it has been associated with leukocyte trafficking and exacerbated autoimmune neuroinflammation [74], as well as *Steap4*, a Th17‐induced effector in CNS‐resident cells that has been implicated in cytokine production, immune cell recruitment, and demyelination [75]. We also observed transcripts that were reduced by injury but increased with anakinra, consistent with a rescuing effect (Figure 8B). These included endothelial‐ and matrix‐associated genes such as *Vwf*, which is reported to be suppressed by IL‐1 signaling in endothelial cells [76] and to play a protective role in maintaining blood–brain barrier integrity [77], as well as structural basement membrane components like *Hmcn1* and *Nid2*, which have been reported to cooperate in preserving barrier linkage and stability [78]. Our analysis also identified two small sets of genes that exhibited amplified transcriptional changes with anakinra (Figure 8C,D). Among those further increased, *Fzd4* has been reported to promote blood–brain barrier stability [79], while *Lyst* is essential for lysosomal trafficking, where its deficiency impairs debris clearance and drives progressive neuroinflammation [80]. In contrast, *Bin2* was further reduced by anakinra, and its suppression has been linked to enhanced phagocytosis [81], suggesting that decreased *Bin2* could complement *Lyst* upregulation in promoting debris clearance. Other transcripts further downregulated by anakinra included *Pf4*, *Socs1*, and *Gpr84*. *Pf4* expression can be induced by IL‐1, TNF, or LPS in monocytes [82], while *Gpr84* is upregulated by IL‐1β, TNFα, or LPS in the brain [83], and *Socs1* is strongly induced by IL‐6 in T cells [84]. Their paradoxical reduction at 6 h post‐injury, despite elevated IL‐1/IL‐6 levels, could reflect counter‐regulatory influences and timing, though residual IL‐1 activity is evident from the additional suppression achieved with anakinra. This interpretation is supported by supplemental RT‐PCR data showing Gpr84 induction by 24 h post‐injury (Figure S2). Taken together, these findings suggest that anakinra selectively reshapes the early transcriptional response to combined injury by attenuating inflammation‐associated transcripts and preserving the expression of certain barrier and matrix genes.

**FIGURE 7:**
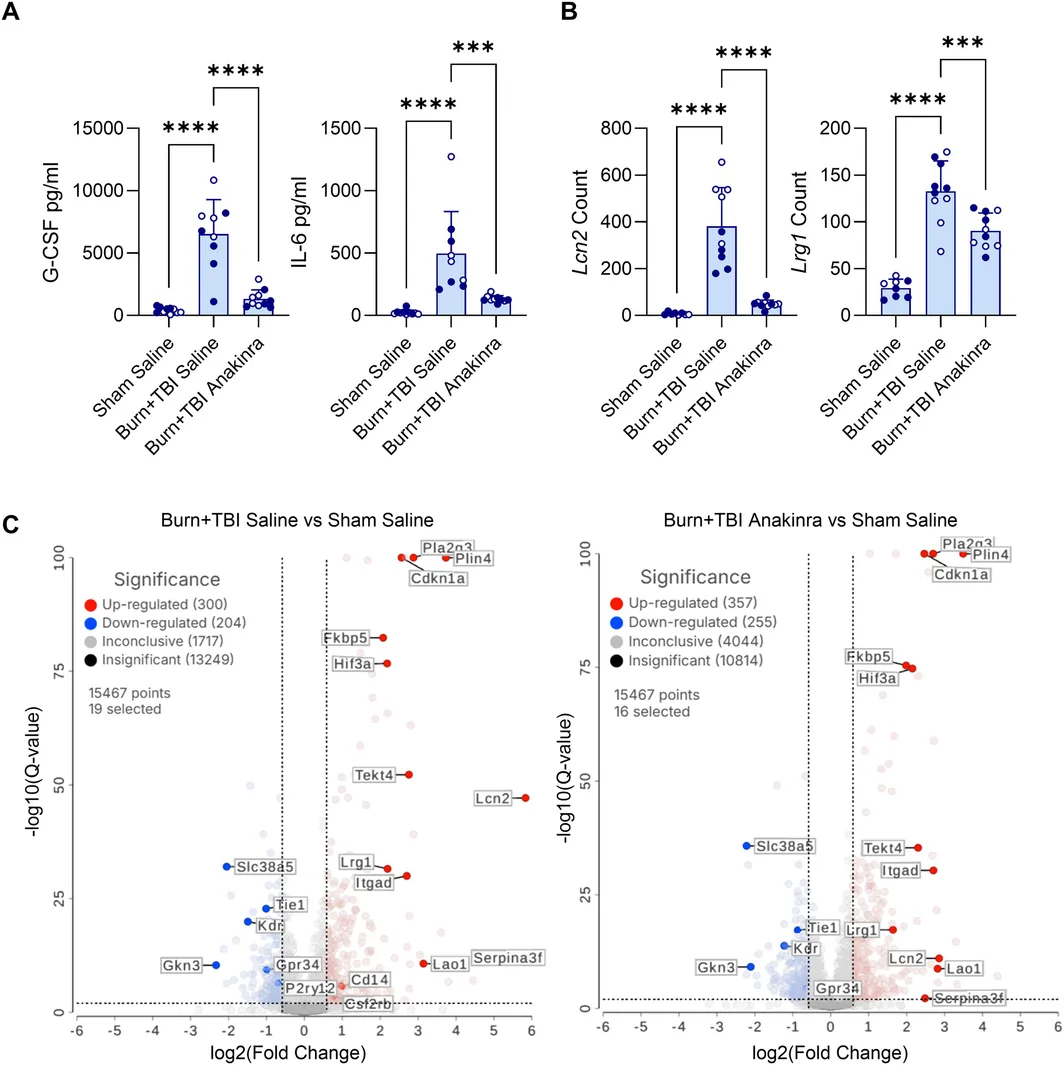
Anakinra attenuates systemic cytokine secretion and selectively modifies brain transcriptional responses to combined injury. Cohorts of male and female mice were administered anakinra immediately after combined injury, then serum and whole‐brain tissue for RNA‐seq were collected at 6 h post‐injury. (A) Serum G‐CSF and IL‐6 quantification plotted as mean ± SD with all points shown; male mice are represented by filled circles and female mice by open circles. (B) Normalized counts for *Lcn2* and *Lrg1* from whole‐brain RNA‐seq, plotted as mean ± SD with all points shown; male and female mice are represented by filled and open circles, respectively. An ANOVA with a post hoc Holm–Sidak test was used for comparisons shown with lines. ***p < 0.001, ****p < 0.0001. (C) Differential expression analysis (volcano plot) of whole‐brain RNA‐seq comparing combined injury groups versus sham (*Q* ≤ 0.01; fold‐change ≥ ±1.5).

**FIGURE 8:**
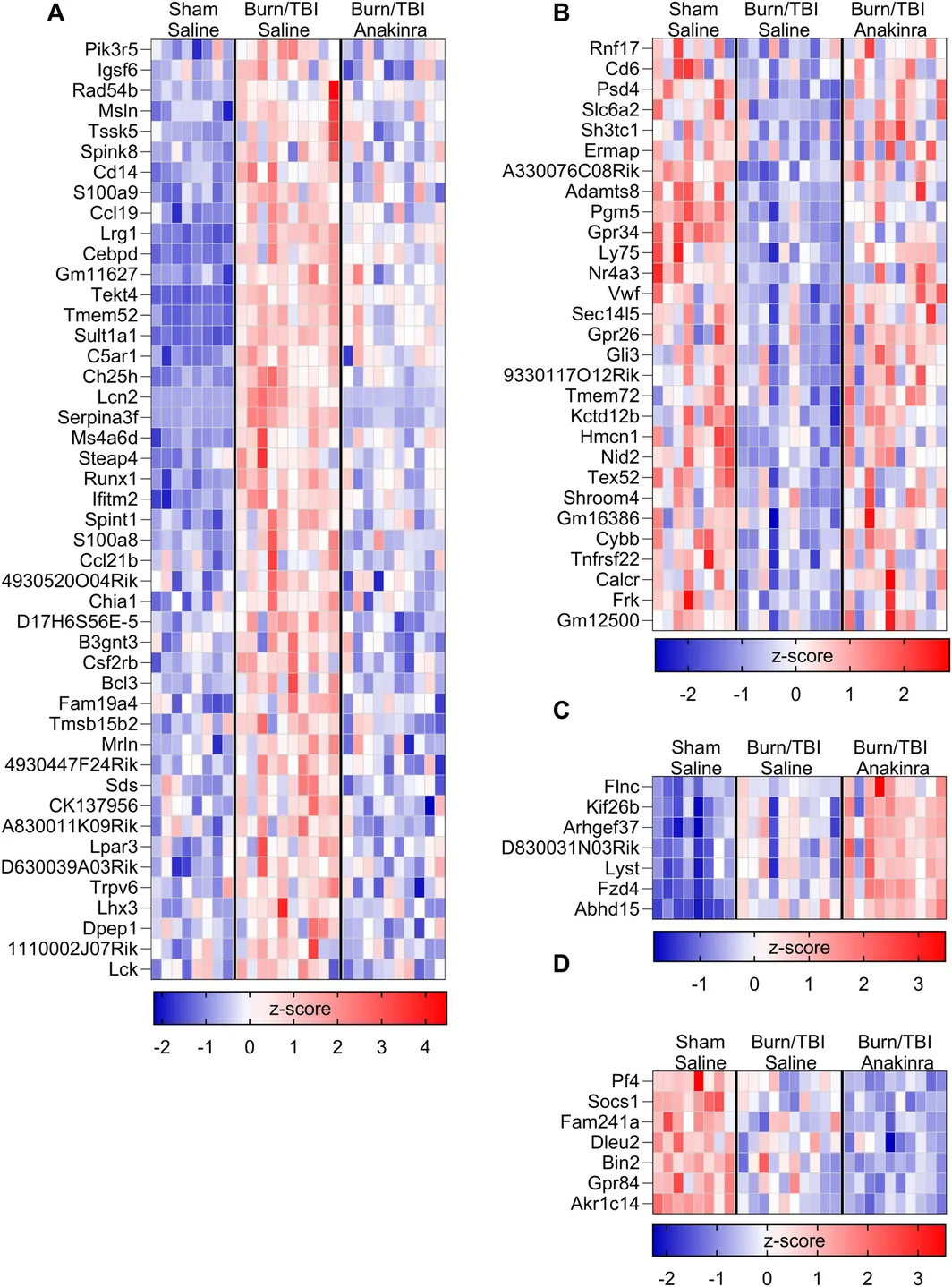
Anakinra modifies expression of inflammatory/immune‐related and barrier‐associated genes following combined injury. To identify transcripts modulated by both injury and anakinra treatment, we applied screening criteria of *Q* ≤ 0.15 and fold‐change ≥ ±1.25. Genes were first required to show differential expression between burn/TBI saline and sham saline and then to meet the same criteria in the burn/TBI anakinra versus burn/TBI saline comparison. Panels show (A) genes increased by injury and attenuated by anakinra, (B) genes reduced by injury and rescued by anakinra, (C) genes further increased by anakinra, and (D) genes further reduced by anakinra.

### 3.7 Cell‐Type Mapping of Bulk DEGs Identifies Broad Transcriptional Engagement of Brain Cell Populations Following Combined Injury

Since prior studies of the brain following burn injury focused largely on isolated features of endothelial or microglial activation and the response to combined injury was unique, we sought to define the broader cellular context of the early brain response to combined injury using single‐nucleus RNA‐seq and projection‐based mapping. To extend our bulk RNA‐seq findings, we performed single‐nucleus RNA‐seq on sham, 6‐h post‐injury, and 24‐h post‐injury brain samples. Clustering analysis identified neuronal and non‐neuronal populations based on a combination of canonical and data‐driven marker genes (Figure 9A). Neuronal nuclei were broadly identified by expression of *Nrg2*, which demonstrated specific expression across neuronal clusters with minimal background in non‐neuronal cells. Inhibitory neurons were identified by *Gad1*, *Gad2*, and *Slc32a1*, while excitatory neurons were marked by *Slc17a7*. A small cluster of Purkinje cells was identified by expression of *Car8*, *Sycp1*, and additional transcripts which have been previously reported as enriched in Purkinje neurons [85]. Other neuronal subtypes were classified based on data‐driven marker genes such as *Gabra6*, *Alk*, *Satb2*, *Meis2*, and *Ntn1*. Non‐neuronal lineages were classified based on the expression of canonical markers for oligodendrocytes, astrocytes, microglia, endothelial cells, pericytes, choroid plexus, and vascular leptomeningeal cells (VLMC). Given the limited number of samples, we applied an injury‐agnostic approach using the tissue_scMappR_custom function to project bulk DEGs onto cell‐type‐specific reference profiles. This function does not model changes in cell‐type abundance or activation state, but it provides a useful framework for predicting the cell types most likely to express transcripts altered by injury. The scMappR analysis predicted diverse cellular contributions to the bulk transcriptional response, with predominant enrichment in vascular and glial lineages (Figure 9B,C). Vascular‐associated nuclei were sparsely represented in our dataset; therefore, gene‐specific localization predictions by scMappR require cautious interpretation (Figures S3 and S4). Nonetheless, the model predicted that the IL‐1‐responsive transcripts *Lcn2* and *Lrg1* were enriched in endothelial cells. To validate the predicted endothelial origin of *Lcn2* expression, we performed immunofluorescence co‐staining with the vascular marker lectin across brain sections, isolated brain microvessels, and liver tissue (Figure 10). Following combined injury, LCN2 was predominantly detected within the vasculature along the midline and within the hypothalamus (Figure 10A). Consistent with this regional heterogeneity, LCN2 staining in isolated brain vasculature was restricted to specific segments, being most prominent in larger vessels (Figure 10B). This vascular‐restricted profile contrasted with the liver, where LCN2 was predominantly detected in hepatocytes (Figure 10C). Together, these findings suggest a broad cellular landscape is activated by combined injury, within which distinct cerebrovascular compartments may exhibit heterogeneous and/or regional molecular responses.

**FIGURE 9:**
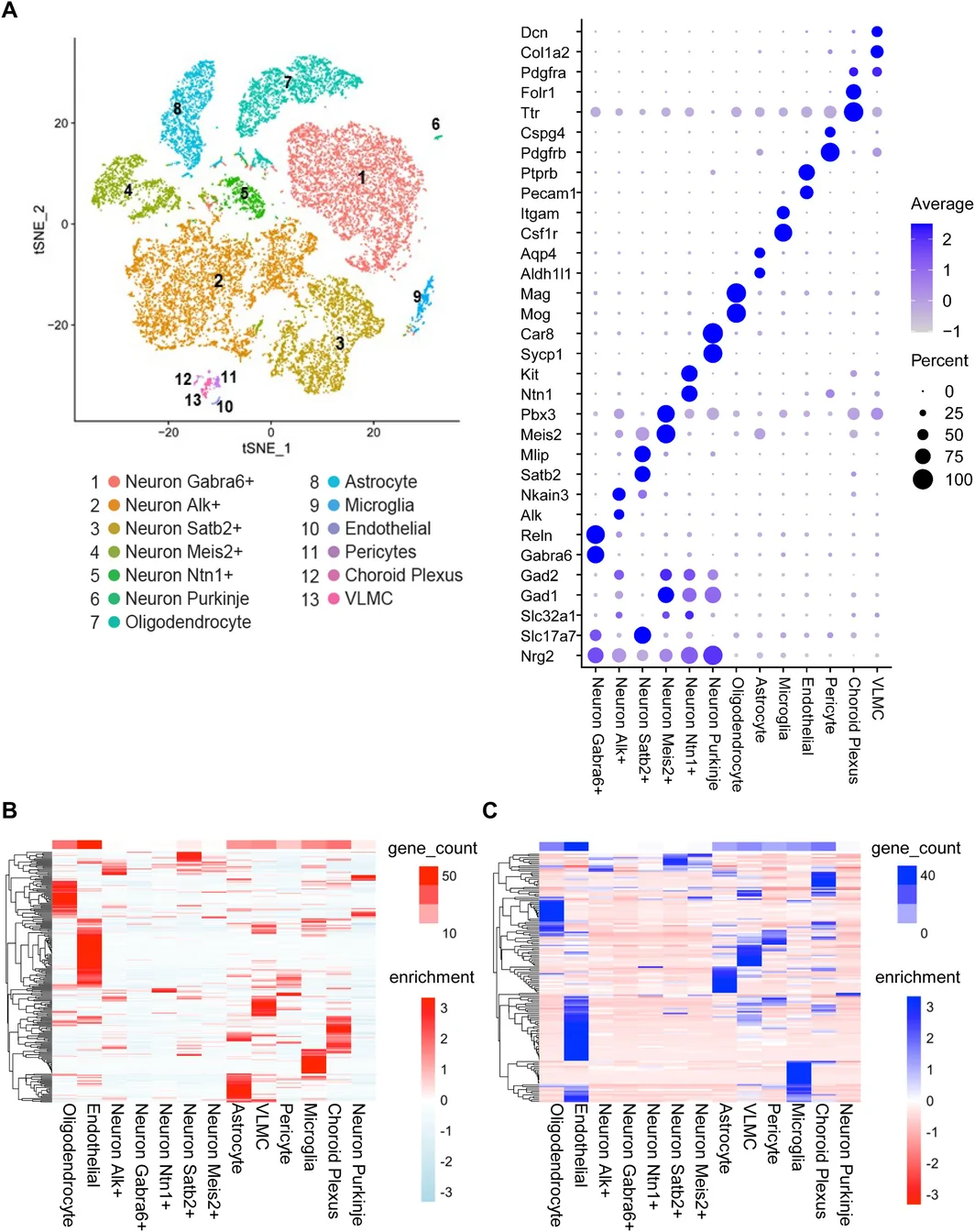
Predicted cell‐type enrichment of bulk RNA‐seq DEGs across diverse brain lineages. Sagittal brain tissue from the ipsilateral hemisphere of female sham, 6 h post‐injury, and 24 h post‐injury mice were used for snRNA‐seq. (A) Thirteen transcriptionally distinct clusters were identified based on canonical and data‐driven marker genes. To infer the cellular origin of transcriptional changes observed in bulk RNA‐seq, an injury‐agnostic projection approach was applied using the tissue_scMappR_custom function from the scMappR package. Bulk RNA‐seq DEGs from the 6 h burn/TBI versus sham comparison (Figure 3A) were input for enrichment analysis. Enrichment of (B) upregulated and (C) downregulated genes within snRNA‐seq clusters are shown as heatmaps, with color scales representing the enriched gene_count for each cluster (top row of heatmap) and enrichment scores for each gene. Full enrichment heat maps with gene names are provided in Figures S3 and S4.

**FIGURE 10:**
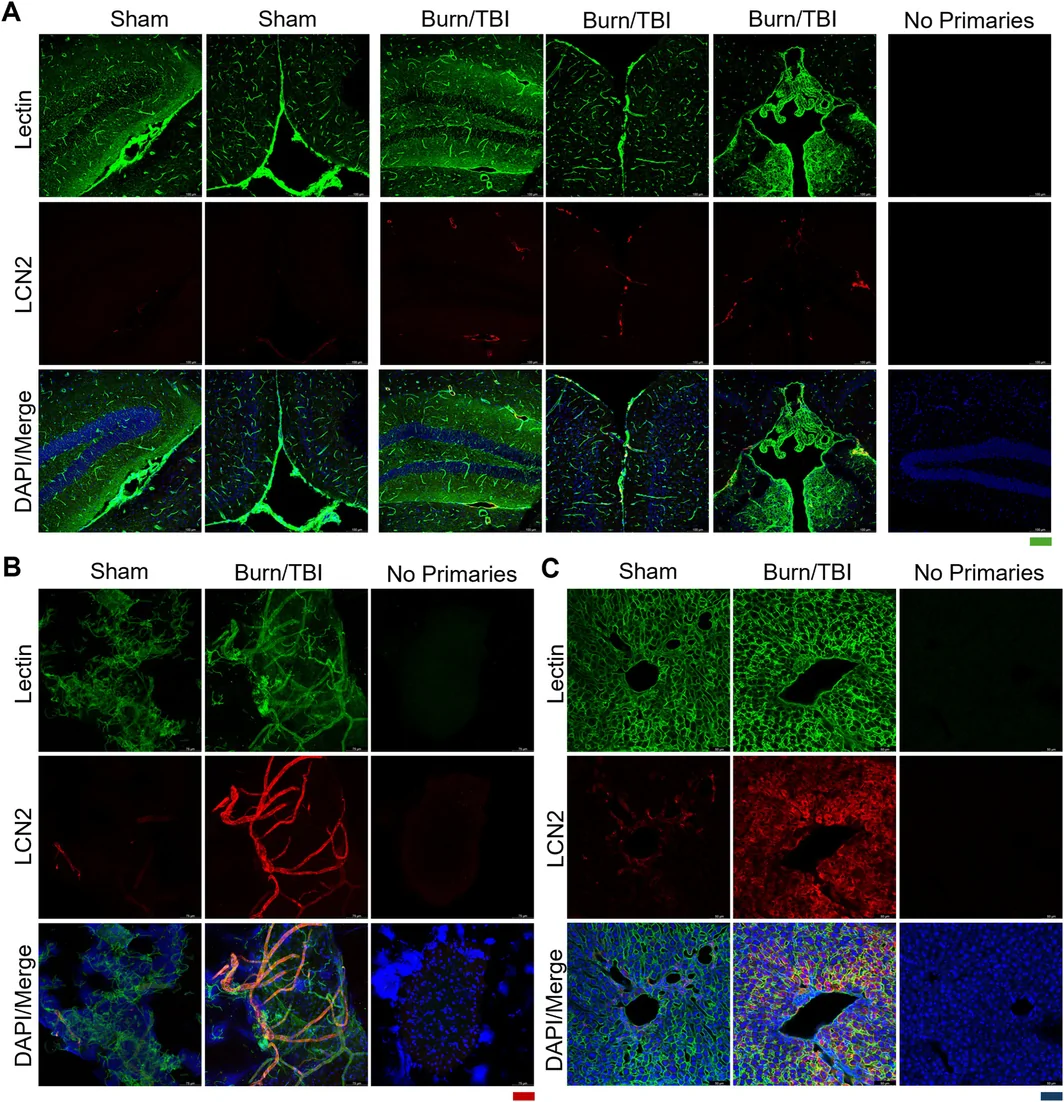
Post‐injury LCN2 localizes to brain vasculature and liver hepatocytes. Brain and liver samples were harvested from mice at 6 h post‐injury to assess LCN2 localization (red), with tomato lectin (green) and DAPI (blue) staining serving to identify blood vessels and nuclei, respectively. All images were captured using a 20× objective lens, with zoom adjusted for each tissue type. (A) Images of coronal brain sections from female Sham and Burn/TBI groups across anatomical regions, including the hippocampus (columns 1, 3), cortical midline fissure (columns 2, 4), and ventricular structures (column 5). Scale bar (green bar) = 100 μm. (B) Isolated brain vasculature from male mice showing heterogenous LCN2 detection across distinct vessel segments following injury. Scale bar (red bar) = 75 μm. (C) Images of liver tissue sections from female mice displaying robust LCN2 detection in hepatocytes post‐injury. Scale bar (blue bar) = 50 μm. Omission of the LCN2 primary antibody and tomato lectin (“No Primaries”) serves as the negative control across all panels.

### 3.8 Combined Injury Induces Distinct Inflammatory and Stress‐Related Gene Profiles Which Are Shared Across the Brain and Liver

Cytokine receptor transcripts that were upregulated in the combined injury bulk RNA‐seq included *Il1r1* and *Il6ra*, and scMappR predicted enrichment of both transcripts in endothelial cells (Figure S3). The *Il6ra* enrichment prediction was not restricted to endothelial cells, but prior studies have shown IL‐6 can directly induce *Lcn2* in the brain endothelium [86] and in hepatocytes [87], suggesting IL‐1‐induced IL‐6 secretion in the combined injury model could be a hierarchical mechanism of inflammatory marker gene induction. We compared therapeutic IL‐1α/β blockade to IL‐6 blockade in the combined injury model to assess a hierarchical relationship for the induction of inflammatory marker genes (*Lcn2* and *Lrg1*) and stress‐responsive transcripts (*Fkbp5*, *Plin4* and *Hif3a*) in the brain (Figure 11A) and liver (Figure 11B). The induction of inflammatory marker genes was at least in part dependent on the IL‐1/IL‐6 axis in both organs, particularly in the liver where IL‐6 blockade markedly attenuated expression. In contrast, stress‐responsive transcripts showed robust induction in both organs that remained elevated despite cytokine blockade. We also examined circulating LCN2 as an inflammatory biomarker and found serum levels were highly dependent on the IL‐1/IL‐6 axis (Figure 11C). While circulating LCN2 levels correlated significantly with *Lcn2* expression in both organs (Figure 11D), the strong linear relationship with hepatic expression was consistent with the liver as a primary source [87]. Overall, these data identify a shared transcriptional response across the brain and liver, implicating circulating mediators. This response features an IL‐1/IL‐6‐driven inflammatory axis alongside a distinct stress‐responsive signature that is consistent with elevated corticosterone levels following combined injury.

**FIGURE 11:**
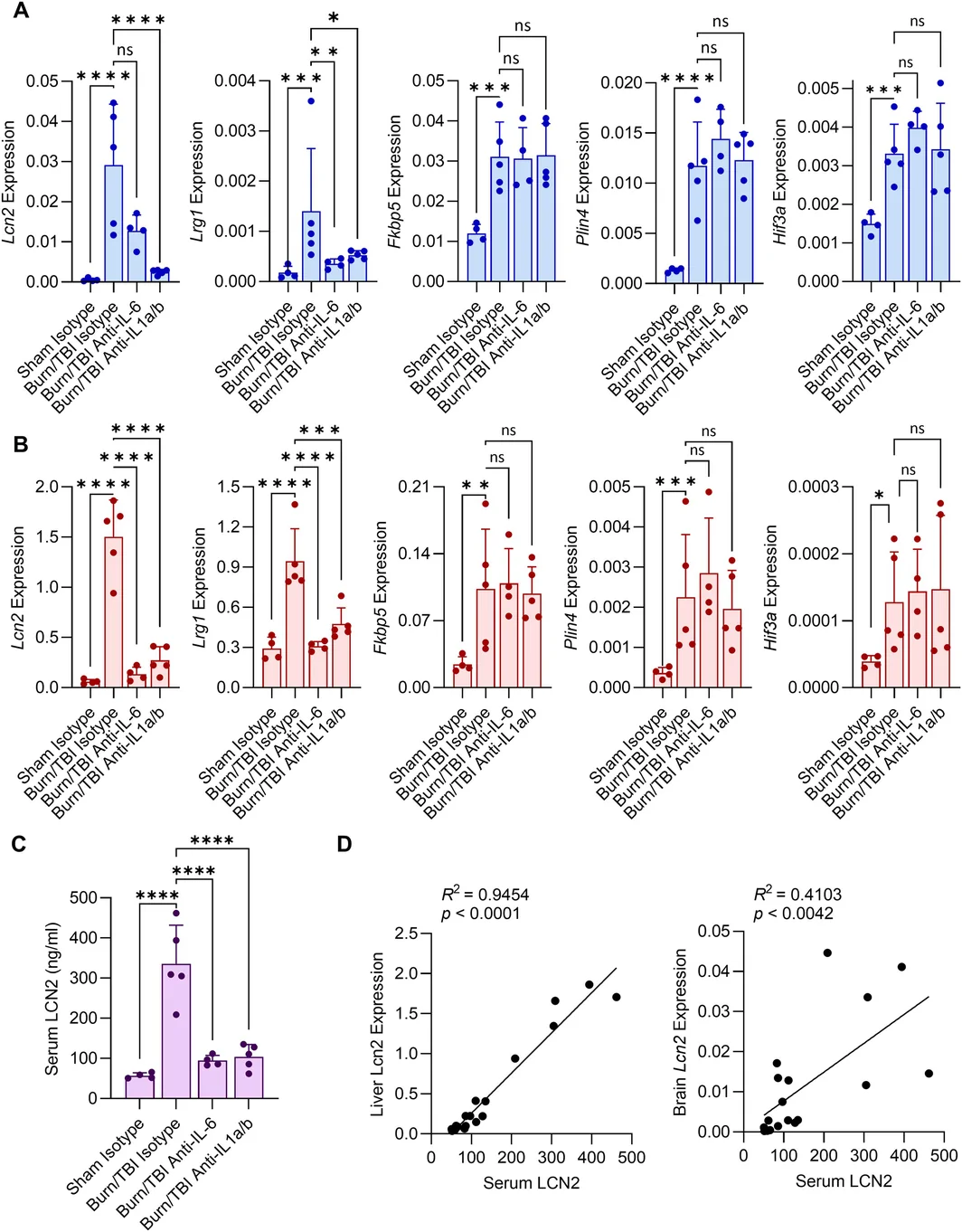
Comparison of IL‐6 and IL‐1α/β blockade reveals a hierarchical inflammatory axis alongside distinct stress‐responsive gene profiles shared across the brain and liver. Female mice were therapeutically administered isotype control, anti‐IL‐6 or combined IL‐1α/β antibodies immediately following injury. Tissues and serum were collected at 6 h post‐injury to evaluate IL‐1 dependent IL‐6 secretion as a hierarchical mechanism that modulates inflammatory marker gene (*Lcn2*, *Lrg1*) or stress‐responsive transcripts (*Fkbp5*, *Plin4*, *Hif3a*) across organs. (A) Whole brain expression. (B) Liver Expression. (C) Serum LCN2 levels quantified by ELISA. (D) Simple linear regression analysis evaluating the relationship between circulating LCN2 protein levels with liver and brain *Lcn2* expression. Expression and serum data are plotted as mean ± SD with all points shown (*n* = 4–5/group). An ANOVA with a post hoc Holm‐Sidak test was used for comparisons shown with lines. *p < 0.05, **p < 0.01, ***p < 0.001, ****p < 0.0001, ns, not significant.

## 4 Discussion

This study introduces a mouse model of concomitant burn and blast‐induced mild TBI to approximate a common combat‐related injury. Both burn and TBI are major clinical concerns with established care guidelines, but these guidelines typically address each injury in isolation, often with competing priorities. The compartmentalized approach to treatment reflects the limited understanding of how the two injuries interact. Mechanistic data on the combined insult are sparse, and to our knowledge only a single prior preclinical study has examined the response [88]. In that work, rats were exposed to a head‐on full body blast followed by a deep partial‐thickness scald burn involving ~10% TBSA and several regions of the brain were profiled with a custom RT‐PCR array (86 genes, 74 analyzable). The report indicated that burn alone elicited a rapid and robust neuroinflammatory response, blast alone produced only modest and delayed changes, and the combined injury condition resulted in a more subtle induction of the selected neuroinflammatory genes than burn alone.

Our model of the combined injury differed from the prior rat study in several ways, including the species used, head‐only blast exposure perpendicular to the blast wave, which allows rapid head movement and associated rotational forces [34], a larger full‐thickness flame burn encompassing 15% TBSA, and a single brief exposure to isoflurane anesthesia rather than the recovery period of 30–60 min between blast and a second anesthetic induction for scald injury used in the rat study. Despite these distinctions and the unbiased transcriptional assessment of the whole ipsilateral brain hemisphere, our results were generally consistent with the prior study in that blast‐induced mild TBI led to very few transcriptional changes in the brain and burn alone induced more remarkable changes; however, in our study the combined injury produced the most robust transcriptional alterations. Cell‐type mapping predicted that these responses involved multiple brain populations, with prominent enrichment within glial and vascular‐associated lineages. Our results also highlight an important asymmetry between systemic and cerebral responses. Burn injury alone was sufficient to induce systemic inflammation, hematopoietic alterations, and robust transcriptional changes within the brain, whereas overlapping blast‐induced mild TBI specifically amplified the transcriptional changes in the brain without further augmenting systemic effects. Together, these findings suggest that the peripheral response to burn plays a pivotal role in sensitizing the brain to mount an amplified transcriptional response within diverse cellular compartments when an additional insult is superimposed.

Gene set enrichment analysis identified glucocorticoid‐responsive signatures in the brain and targeted expression profiling of the liver revealed a shared induction of stress‐responsive genes across organs, consistent with the increase of corticosterone following combined injury. Although the present study does not directly test whether HPA‐axis activation is required for these transcriptional changes, the concordance between CRF‐blockade‐derived transcriptional signatures, increased circulating corticosterone, and cytokine‐blockade‐insensitive stress‐responsive gene induction is consistent with endocrine stress signaling as a contributor to the early multi‐organ response. Since our study was limited to an early transcriptional snapshot associated with corticosterone elevation, we cannot attribute benefits or harm to activation of the HPA‐axis. However, prior burn literature indicates that acute cortisol release is necessary for maintaining hemodynamic stability and mounting an adequate stress response, yet persistent hypercortisolemia can drive catabolism, immune suppression, and increased infection risk, and in some patients adrenal insufficiency may develop and is associated with greater mortality [59, 60, 61]. This aligns with the broader view that glucocorticoids play an important role in restraining early inflammatory cascades but become deleterious when sustained [89]. The HPA signature we observed in the combined injury model may therefore reflect an acutely beneficial response, as prior stress and glucocorticoid exposure have been shown to diminish subsequent HPA activity and sensitize both peripheral and neuroinflammatory cytokine production, amplifying responses to a later challenge [90, 91, 92, 93].

In addition to the HPA‐associated signature revealed by our enrichment results, we also observed enrichment of LPS response‐related gene sets, characterized by concordant regulation of a broad inflammatory response, along with negative enrichment of multiple vascular and endothelial signatures. We assessed IL‐1R1 signaling as a central mediator of systemic inflammation and cerebral responses following combined injury based on our prior report which identified a key role for the pathway in driving post‐burn inflammation and anemia [32]. In the combined injury setting, both IL‐1α/β neutralization and anakinra blunted systemic cytokine secretion and attenuated an induction of inflammatory marker genes, *Lcn2* and *Lrg1*, in the brain. An unbiased transcriptomic analysis of whole brain tissue indicated anakinra selectively reshaped expression patterns, generally reflecting attenuation of inflammation‐associated transcripts and preservation of certain barrier and matrix genes rather than normalizing the global response to injury. This result, along with the heterogeneous localization of LCN2 within vascular structures of the brain, suggests that the IL‐1‐mediated inflammatory response may be compartmentalized in the acute period following injury. Furthermore, the induction of inflammatory marker genes across both the brain and liver is, at least in part, dependent on IL‐1‐induced IL‐6 secretion, reinforcing the coordinated multi‐organ nature of the systemic response. Given that LCN2 localizes to vascular structures in the brain but to parenchymal hepatocytes in the liver, these tissue‐specific responses likely reflect which distinct cell types express the necessary receptors and the degree of physical accessibility permitted by local vascular barriers. While these data only capture an acute snapshot of combined injury, they demonstrate a capacity for IL‐1‐targeted therapies to selectively modulate neuroinflammatory transcription.

Finally, our observation that anakinra attenuates hematopoietic changes driving anemia after combined injury is especially relevant given greater anemia severity has been linked to poor neurological recovery following TBI [20] and recent trials suggest improved outcomes when hemoglobin is maintained at ≥ 9–10 g/dL [21, 22]. Although the American Burn Association guidelines permit lower hemoglobin thresholds, they acknowledge that such restrictive practices may not be appropriate for burn patients with concomitant brain injury [23]. This caveat is difficult to interpret in light of a growing recognition that burn injury itself provokes neuroinflammation and secondary brain dysfunction [3], suggesting that many major burn patients could face some degree of cerebral vulnerability. This perspective underscores the need to integrate neurological assessments into future evaluations of transfusion thresholds, explore the utility of brain injury biomarkers in the burn setting as guides for transfusion strategies, and prioritize upstream interventions. By blunting inflammation‐induced suppression of erythropoiesis, therapies such as anakinra may reduce transfusion requirements, preserve hemoglobin above vulnerable levels, and mitigate neurologic risk in patients with combined injuries.

In conclusion, this study provides the most comprehensive analysis of concomitant burn and blast‐induced mild TBI to date, encompassing systemic profiling of cytokine secretion and hematopoietic changes, unbiased transcriptomic analysis of brain responses to single and combined injuries, therapeutic assessment of IL‐1 blockade, and integration with single‐nucleus data to predict the cell types engaged. Across these analyses, burn injury emerged as the predominant driver of systemic inflammation and impaired erythropoiesis, whereas overlapping TBI amplified cerebral transcriptional programs. IL‐1 signaling was identified as a tractable pathway associated with both systemic and cerebral responses, with blockade attenuating cytokine secretion, hematopoietic alterations, and select inflammation‐associated brain transcripts. Together, these findings define an early immune‐endocrine transcriptional response to combined burn and blast‐induced mild TBI, while longer‐term neurological outcomes, histological injury, and BBB integrity remain important areas for future investigation.

## Author Contributions

J.C.G. and M.J.R. designed and acquired funding for the study. J.C.G., M.J.R., and H.C.S. drafted the manuscript. All authors analyzed data and edited the manuscript.

## Funding

This work was supported by the Office of the Assistant Secretary of Defense for Health Affairs through the Military Burn Research Program (W81XWH‐22‐1‐0849), National Institutes of Health (NIH) (RO1HL155579 and R01NS137887).

## Conflicts of Interest

The authors declare no conflicts of interest.

## Supporting information


**Figure S1:** Male Ter119+ bone marrow composition and blood cell counts overlaid on female data from Figure 2 main text. Male mice (*n* = 5/gp) were subjected to sham, TBI, burn or burn/TBI with bone marrow cells and blood collected on day 7 post injury for analysis. (A) Flow cytometry analysis for Ter119+ proportion of the bone marrow. (B) Red blood cell and (C) white blood cell values determined on an automated hematology instrument. All plots show male values (filled circles) overlaid with female values (empty circles) from Figure 2 main text and line at mean. Plots are provided to show general concordance of the results without statistical comparison.

**Figure S2:** Gpr84 expression is increased in the brain 24 h after mice are subjected to burn or combined injury. Ipsilateral half brain tissue was isolated at 24 h post‐injury from cohorts of male and female mice. RNA processing and SYBR Green based RT‐PCR followed methodology described in the main text using primers for Gpr84 (forward 5′‐CTGAAGACAAGTGTGAAAACTGG‐3′, reverse 5′ GATAGCCCAACACAGACTCAT‐3′) and Actb (forward 5′‐GACTCATCGTACTCCTGCTTG‐3′, reverse 5′‐GATTACTGCTCTGGCTCCTAG‐3′). Gpr84 expression levels for males (filled circle) and females (open circle) are plotted as mean ± SD. An ANOVA with a post hoc Holm–Sidak test was used for comparisons shown with lines. **p < 0.01, ***p < 0.001, ns, not significant.

**Figure S3:** Expanded scMappR heatmap for upregulated genes.

**Figure S4:** Expanded scMappr heatmap for downregulated genes.

**Table S1:** RT‐PCR primer sequences.

**Data S1:** Supporting Information.

**Data S2:** Supporting Information.

**Data S3:** Supporting Information.

**Text S1:** Supporting Information.

## Data Availability

RNA sequencing datasets are available in the NCBI Gene Expression Omnibus under accession numbers GSE308043 (bulk RNA‐Seq) and GSE308044 (single‐nucleus RNA‐Seq).
