# Supplementary material for: Multi‐Organ Inflammatory and Endocrine Crosstalk Shapes the Early Brain Response to Combined Burn and Blast Injury
Source: FASEB J. 2026 Oct 29;40(21):e72354. doi: 10.1096/fj.202504681R (PMC13717289; doi:10.1096/fj.202504681R)
Supplement: Supplementary file 1 [file FSB2-40-e72354-s001.pdf]

Supplemental RNA-seq QC Analysis

Supplemental RNA-seq QC Analysis Figure 1. Multiple Injury Bulk RNA-seq Analysis

Estimated read counts (numReads) from Salmon outputs were used to generate a count matrix in Partek Flow (Illumina). Features with fewer than 10 counts in at least 50% of samples were filtered out and samples were normalized using the median ratio method. Principle component analysis (PCA) was performed for QC. (A) Prior to batch correction, QC analyses indicated the presence of a batch effect, consistent with male and female samples from separate experiments which were processed and sequenced in separate runs. (B) After batch correction, four samples were identified as outliers with distinct clustering. The outliers were associated with increased (C) Rn45s expression and (D) increased ribosomal protein gene expression which was attributed to technical artifact. (E) PCA confirmed the effectiveness of batch and technical artifact correction. Therefore, differential expression analysis was performed in DESeq2 with the design formula ~ *batch* + *artifact* + *condition*. For visualizations and GSEA, median ratio normalized counts were adjusted by adding a pseudocount of +1 followed by batch and artifact correction using the general linearized model in Partek Flow.

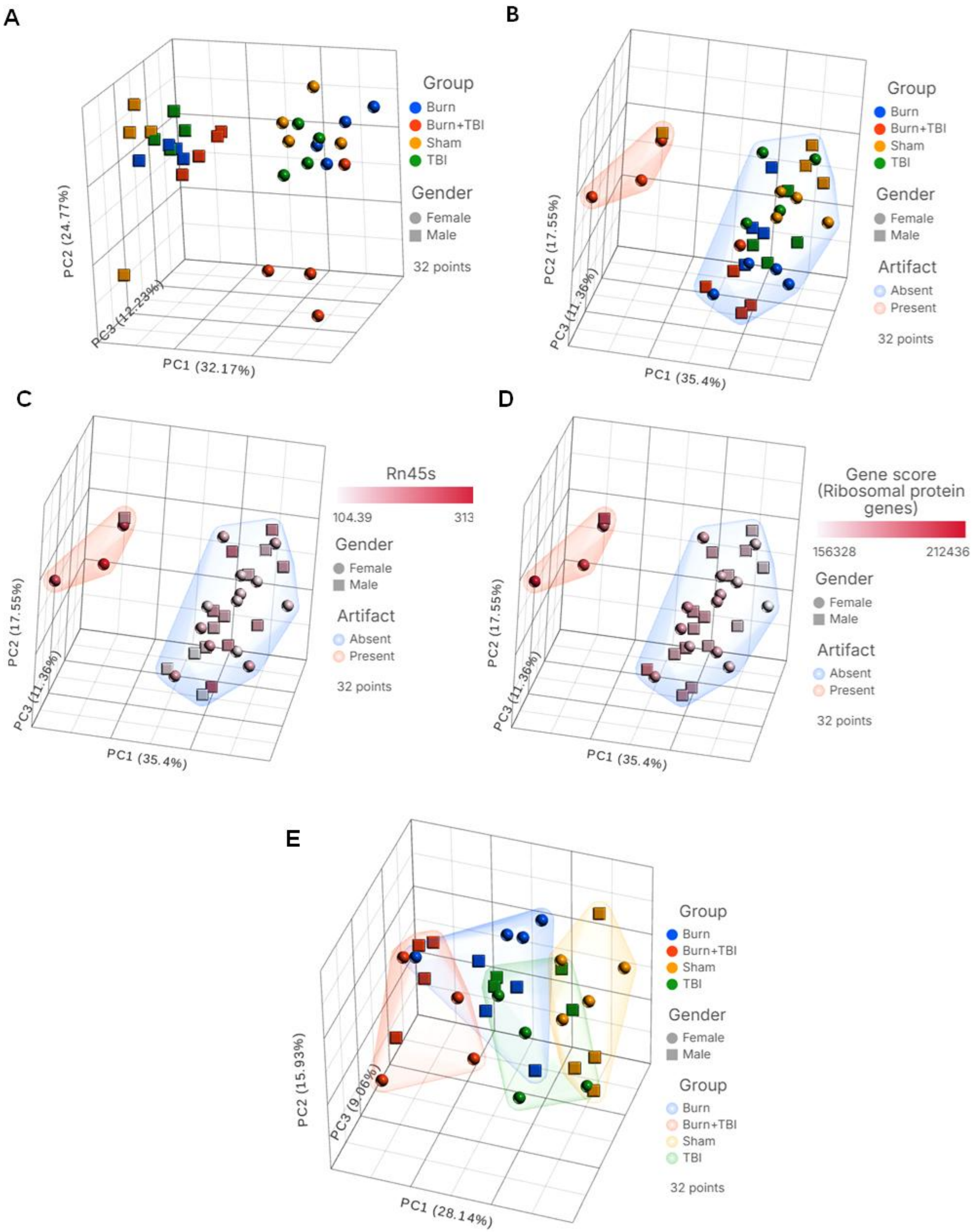
A
Group
Burn
Burn+TBI
Sham
TBI
Gender
Female
Male
32 points
PC2 (24.77%)
PC3 (12.23%)
PC1 (32.17%)
B
Group
Burn
Burn+TBI
Sham
TBI
Gender
Female
Male
Artifact
Absent
Present
32 points
PC2 (17.55%)
PC3 (11.36%)
PC1 (35.4%)
C
Rn45s
104.39
313
Gender
Female
Male
Artifact
Absent
Present
32 points
PC2 (17.55%)
PC3 (11.36%)
PC1 (35.4%)
D
Gene score (Ribosomal protein genes)
156328
212436
Gender
Female
Male
Artifact
Absent
Present
32 points
PC2 (17.55%)
PC3 (11.36%)
PC1 (35.4%)
E
Group
Burn
Burn+TBI
Sham
TBI
Gender
Female
Male
Group
Burn
Burn+TBI
Sham
TBI
32 points
PC2 (15.93%)
PC3 (9.06%)
PC1 (28.14%)

Supplemental RNA-seq QC Analysis Figure 2. Anakinra Bulk RNA-seq Analysis

Estimated read counts (numReads) from Salmon outputs were used to generate a count matrix in Partek Flow. Features with <10 counts in $\geq 80\%$ of samples were removed and samples were normalized using the median ratio method. (A) PCA prior to correction demonstrated good clustering by injury and a subtle batch effect between genders, consistent with male and female studies performed separately. (B) PCA after batch correction demonstrated clustering by biological condition. Therefore, differential expression was modeled in DESeq2 with the design formula ~ *batch* + *condition*. Median ratio normalized counts for visualizations were adjusted by adding a pseudocount of +1 followed by batch correction using the general linearized model in Partek Flow.

A

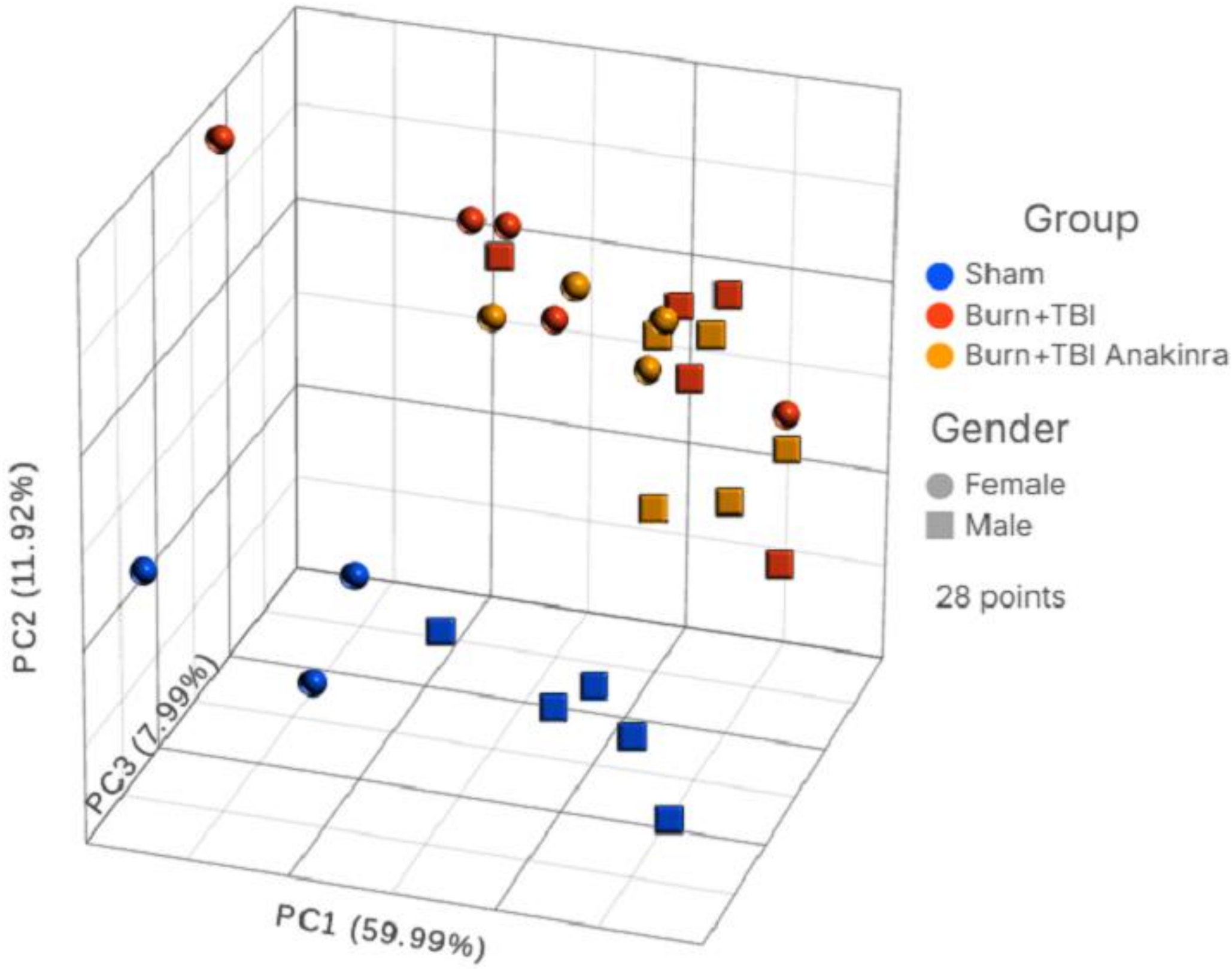
Group
Sham
Burn+TBI
Burn+TBI Anakinra
Gender
Female
Male
28 points
PC2 (11.92%)
PC3 (7.99%)
PC1 (59.99%)

B

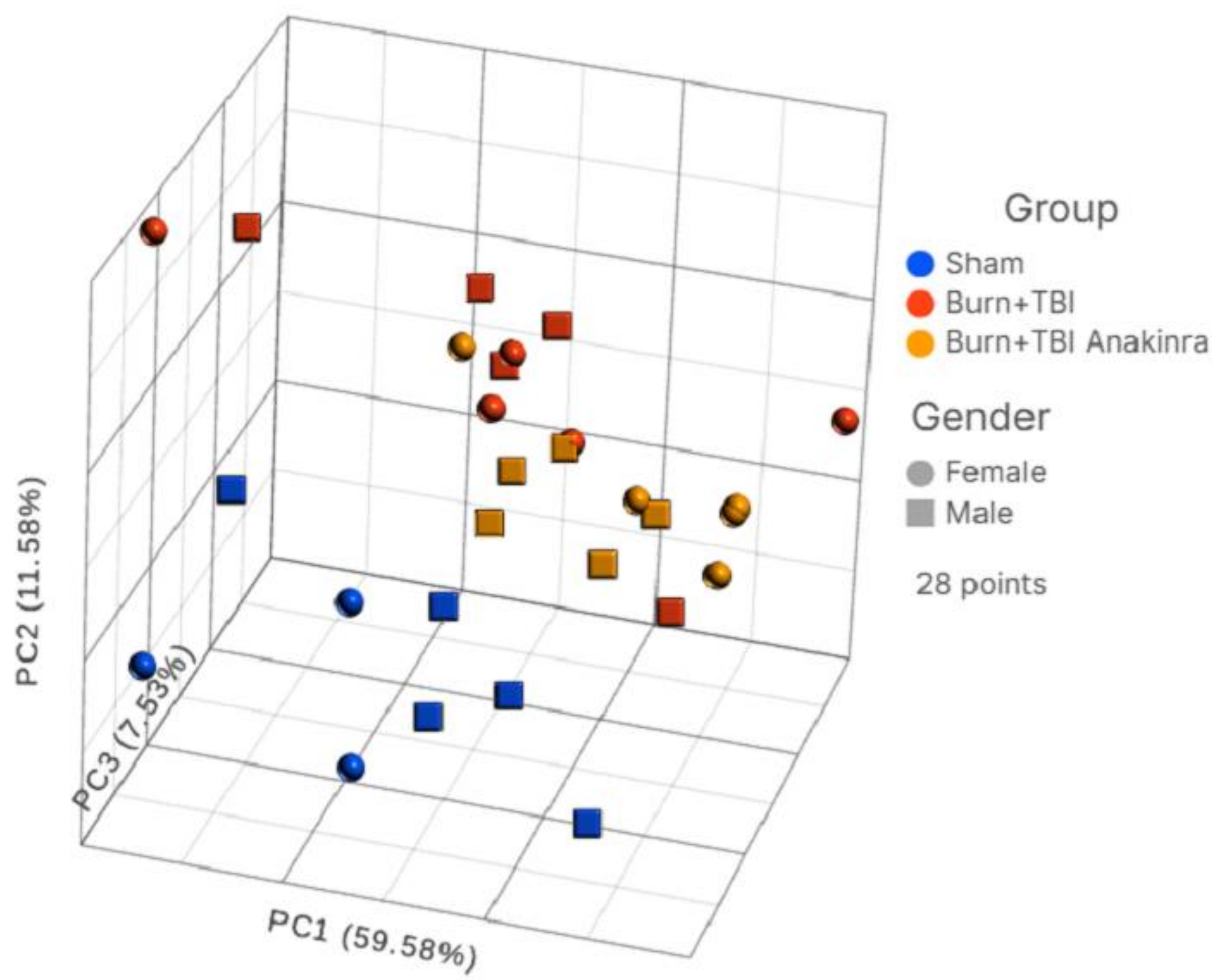
Group
Sham
Burn+TBI
Burn+TBI Anakinra
Gender
Female
Male
28 points
PC2 (11.58%)
PC3 (7.53%)
PC1 (59.58%)
