# Supplementary material for: Multi‐Organ Inflammatory and Endocrine Crosstalk Shapes the Early Brain Response to Combined Burn and Blast Injury
Source: FASEB J. 2026 Oct 29;40(21):e72354. doi: 10.1096/fj.202504681R (PMC13717289; doi:10.1096/fj.202504681R)

Supplemental Figure 1. Male Ter119+ bone marrow composition and blood cell counts overlaid on female data from figure 2 main text. Male mice (n = 5/gp) were subjected to sham, TBI, burn or burn/TBI with bone marrow cells and blood collected on day 7 post injury for analysis. (**A**) Flow cytometry analysis for Ter119+ proportion of the bone marrow. (**B**) Red blood cell and (**C**) white blood cell values determined on an automated hematology instrument. All plots show male values (filled circles) overlaid with female values (empty circles) from figure 2 main text and line at mean. Plots are provided to show general concordance of the results without statistical comparison.

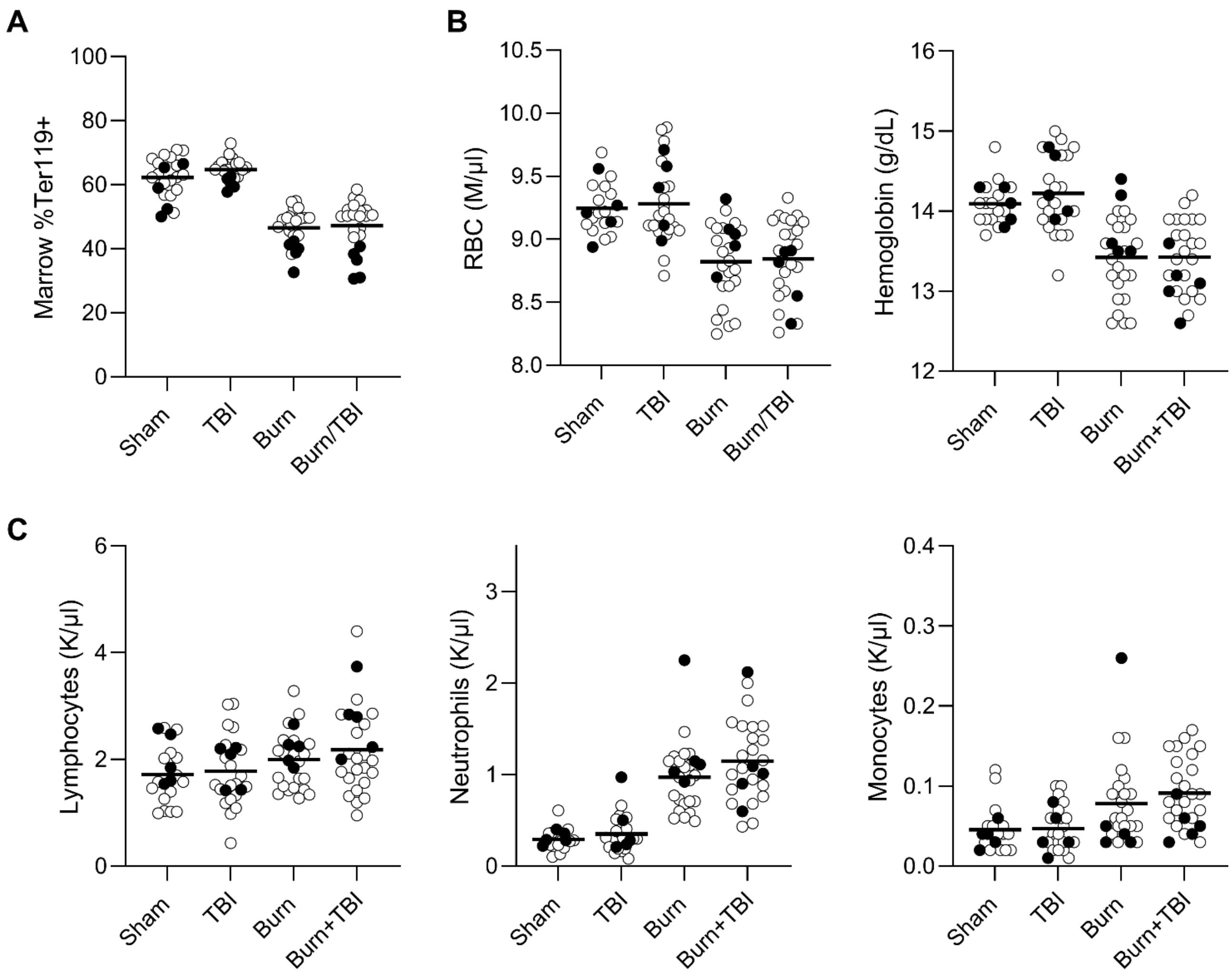

Supplement: Supplementary file 2 [file FSB2-40-e72354-s005.pdf]
