# Supplementary material for: Multi‐Organ Inflammatory and Endocrine Crosstalk Shapes the Early Brain Response to Combined Burn and Blast Injury
Source: FASEB J. 2026 Oct 29;40(21):e72354. doi: 10.1096/fj.202504681R (PMC13717289; doi:10.1096/fj.202504681R)

Supplemental Figure 4.
Expanded scMappr heatmap for downregulated genes.

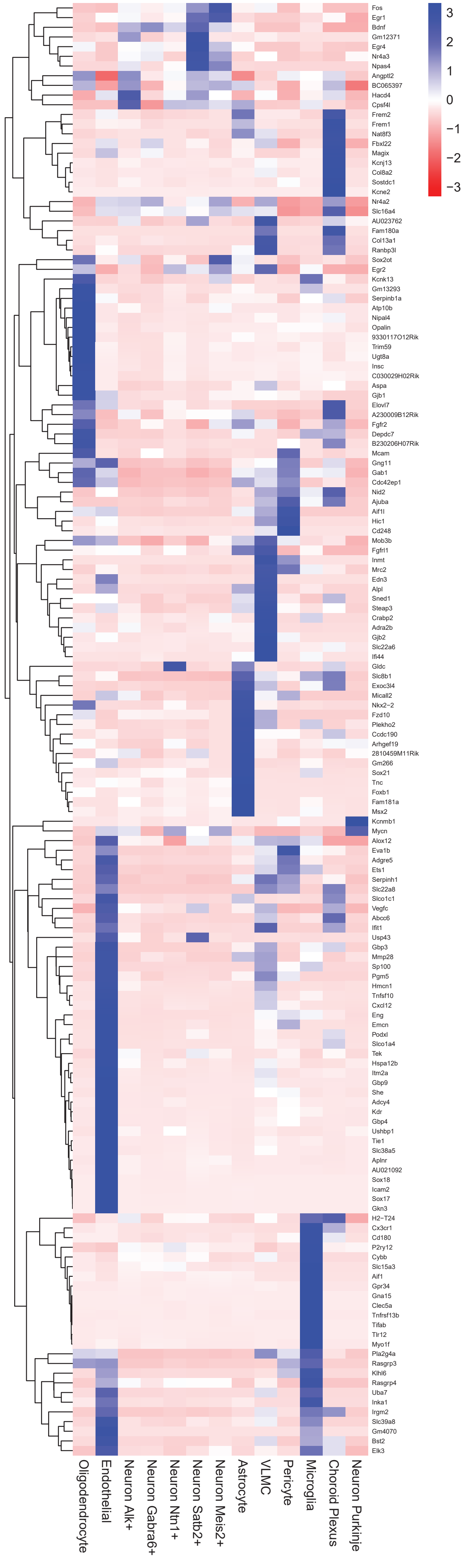

Supplement: Supplementary file 3 [file FSB2-40-e72354-s007.pdf]
