# Supplementary material for: Multi‐Organ Inflammatory and Endocrine Crosstalk Shapes the Early Brain Response to Combined Burn and Blast Injury
Source: FASEB J. 2026 Oct 29;40(21):e72354. doi: 10.1096/fj.202504681R (PMC13717289; doi:10.1096/fj.202504681R)

Supplemental Figure 2. ***Gpr84*** **expression is increased in the brain 24 hours after mice are subjected to burn or combined injury.** Ipsilateral half brain tissue was isolated at 24 h post-injury from cohorts of male and female mice. RNA processing and SYBR Green based RT-PCR followed methodology described in the main text using primers for Gpr84 (forward 5’-CTGAAGACAAGTGTGAAAACTGG-3′, reverse 5’-GATAGCCCAACACAGACTCAT-3’) and Actb (forward 5’-GACTCATCGTACTCCTGCTTG-3′, reverse 5′-GATTACTGCTCTGGCTCCTAG-3′). Gpr84 expression levels for males (filled circle) and females (open circle) are plotted as mean ± SD. An ANOVA with a post hoc Holm–Sidak test was used for comparisons shown with lines. **P < 0.01, ***P < 0.001.

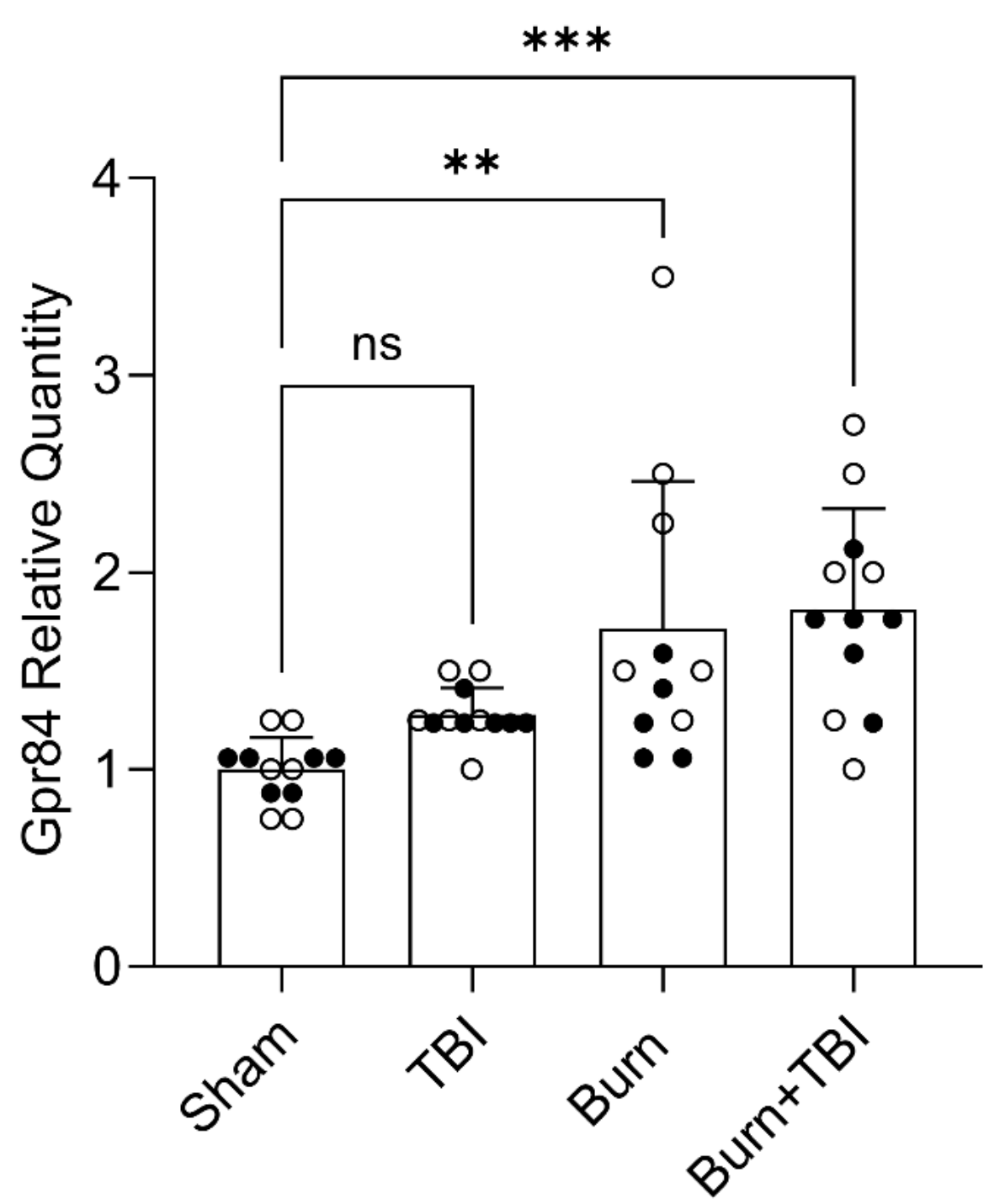

Supplement: Supplementary file 5 [file FSB2-40-e72354-s009.pdf]
