# Supplementary material for: Multi‐Organ Inflammatory and Endocrine Crosstalk Shapes the Early Brain Response to Combined Burn and Blast Injury
Source: FASEB J. 2026 Oct 29;40(21):e72354. doi: 10.1096/fj.202504681R (PMC13717289; doi:10.1096/fj.202504681R)
Supplement: Supplementary file 6 [file FSB2-40-e72354-s002.docx]

| Supplemental Table 1. RT-PCR Primer Sequences | | |
| --- | --- | --- |
| Genes | Forward (5'->3') | Reverse (5'->3') |
| *Actb* | GACTCATCGTACTCCTGCTTG | GATTACTGCTCTGGCTCCTAG |
| *Lcn2* | TCTGGGAAATATGCACAGGTATC | ATGGCGAACTGGTTGTAGTC |
| *Lrg1* | GAGGACAGACATAGAGGAGCA | CAGTATCAGGCATTCCTTGAGG |
| *Fkbp5* | GACACCAAAGAAAAGCTGACG | ACTATCTTCCTGTACTGAATCACG |
| *Hif3a* | GTATCATCTGCGTCCACTTCC | GTATGTTGCTCCGTTTGTTCC |
| *Plin4* | GACTTACAAACAGCAACAGACC | AAACTTCCCATGTCCTTGTCT |
